# An integrated model for termination of RNA polymerase III transcription

**DOI:** 10.1126/sciadv.abm9875

**Published:** 2022-07-13

**Authors:** Juanjuan Xie, Umberto Aiello, Yves Clement, Nouhou Haidara, Mathias Girbig, Jana Schmitzova, Vladimir Pena, Christoph W. Müller, Domenico Libri, Odil Porrua

**Affiliations:** ^1^ Université Paris Cité, CNRS, Institut Jacques Monod, F-75013 Paris, France.; ^2^ European Molecular Biology Laboratory (EMBL), Structural and Computational Biology Unit, 69117 Heidelberg, Germany.; ^3^ Joint PhD degree from EMBL and Heidelberg University, Faculty of Biosciences, Heidelberg, Germany.; ^4^ Max Planck Institute for Biophysical Chemistry, Macromolecular Crystallography, Am Fassberg 11, 37077 Goettingen, Germany.

## Abstract

RNA polymerase III (RNAPIII) synthesizes essential and abundant noncoding RNAs such as transfer RNAs. Controlling RNAPIII span of activity by accurate and efficient termination is a challenging necessity to ensure robust gene expression and to prevent conflicts with other DNA-associated machineries. The mechanism of RNAPIII termination is believed to be simpler than that of other eukaryotic RNA polymerases, solely relying on the recognition of a T-tract in the nontemplate strand. Here, we combine high-resolution genome-wide analyses and in vitro transcription termination assays to revisit the mechanism of RNAPIII transcription termination in budding yeast. We show that T-tracts are necessary but not always sufficient for termination and that secondary structures of the nascent RNAs are important auxiliary cis-acting elements. Moreover, we show that the helicase Sen1 plays a key role in a fail-safe termination pathway. Our results provide a comprehensive model illustrating how multiple mechanisms cooperate to ensure efficient RNAPIII transcription termination.

## Teaser

Nucleic acid signals and an extrinsic termination factor cooperate to ensure efficient RNAPIII transcription termination.

## INTRODUCTION

Transcription termination is an essential process that sets the borders between genes, therefore avoiding interference between neighboring transcription units. Furthermore, transcription termination plays an important role in the maintenance of genome integrity by limiting the possible conflicts between transcribing RNA polymerases (RNAPs) and other cellular machineries involved in DNA replication or repair [reviewed in (*1*)].

Transcription termination can be envisioned as a multistep process consisting of the recruitment of termination factors, the recognition of sequence motifs, RNAP pausing, and, lastly, the release of RNAP and the transcript from the DNA. This last step involves a remodeling of an intricate network of interactions between RNAP, the nascent RNA, and the DNA template [reviewed in (*2*)]. Within this network, the interactions between the polymerase and the RNA:DNA hybrid are considered as the main determinant of stability of the elongation complex (EC) (*3*). Most eukaryotic organisms have three different RNAPs that are specialized in producing different classes of transcripts and seem to adopt different strategies to efficiently terminate transcription. RNAPI is responsible for the synthesis of ribosomal RNAs (rRNAs), RNAPII transcribes all protein-coding genes and several classes of noncoding genes, and RNAPIII synthetizes short and abundant transcripts among which are all tRNAs, the 5*S* rRNA, and several additional noncoding RNAs.

The mechanisms of transcription termination of the three polymerases have been extensively characterized in the eukaryotic model *Saccharomyces cerevisiae*, and many of the principles uncovered in this organism seem to be highly conserved from yeast to humans [reviewed in (*2*)]. RNAPI and RNAPII require extrinsic protein factors to terminate transcription. RNAPI pauses when it encounters a Myb-like factor bound to the DNA downstream of each rRNA gene (*4*, *5*). The release of the paused RNAPI is then mediated by additional proteins, specifically the Rat1 exonuclease and the helicase Sen1 (*6*, *7*), which are also major termination factors for RNAPII (see below).

The mechanism of RNAPII transcription termination is more complex and involves the action of a larger number of proteins. There are two major termination pathways for RNAPII [reviewed in (*1*)]. Transcription termination at protein-coding genes relies on a multisubunit complex that is responsible for the cotranscriptional cleavage of the pre-mRNA at the polyadenylation site and the addition of a poly(A) tail. The downstream portion of the nascent transcript is then targeted by Rat1 (XRN2 in humans), which degrades the RNA molecule until it encounters RNAPII and promotes its release from the DNA (*8*–*12*).

The second pathway is devoted to termination of noncoding transcription and plays an essential role in the control of pervasive transcription and in the biogenesis of small nucleolar RNAs (*1*, *13*). This pathway depends on a complex composed of two RNA binding proteins, Nrd1 and Nab3, and the aforementioned helicase Sen1 (i.e., the NNS complex). Whereas Nrd1 and Nab3 recognize specific sequence motifs that are enriched in the target noncoding RNAs, the helicase Sen1 induces the dissociation of the EC (*14*–*18*). The mechanisms of action of Sen1 in RNAPII transcription have been extensively characterized at the molecular level by our group and others (*14*, *19*–*22*). Briefly, Sen1 uses the energy of adenosine 5′-triphosphate (ATP) hydrolysis to translocate along the nascent RNA toward the transcribing RNAPII, and upon transcriptional pausing, it collides with the polymerase and induces its dissociation from the DNA.

A large body of evidence supports the notion that, in contrast to the other RNAPs, RNAPIII can terminate precisely and efficiently at a particular DNA sequence without the need for accessory proteins [reviewed in (*2*, *23*)]. A typical RNAPIII terminator consists of a stretch of thymidines (Ts) of variable length in the nontemplate DNA strand that, according to the current model, is sufficient to promote both pausing and release of RNAPIII. Upon transcription of a T-tract, the weakness of the resulting rU:dA hybrid is thought to be central to the destabilization of the RNAPIII EC (*24*). The particular sensitivity of RNAPIII to weak rU:dA hybrids compared to other RNAPs that do not sense T-tracts as terminators is believed to depend on the less-extensive interactions between RNAPIII and the RNA:DNA hybrid (*25*). The Ts in the nontemplate strand play an additional critical role in transcription termination (*26*), as they have been proposed to be recognized by the C37 and C53 subunits of RNAPIII that also contribute to termination (*27*, *28*). An alternative model proposed by Nielsen and coauthors (*29*) posits that T-tracts are required for RNAPIII pausing but are not sufficient for its release from the DNA. These authors have proposed that the folding of the nascent RNA into a hairpin-like structure in the vicinity of the paused RNAPIII is an absolute requirement for termination. The hairpin would invade the RNA exit channel of the polymerase, thus provoking its dissociation from the DNA. The proposed mechanism is reminiscent of the so-called intrinsic termination pathway described for bacterial RNAP. This hairpin-dependent model remains, however, highly controversial since it is seemingly in disagreement with a large body of former experimental evidence (*30*).

The model according to which sequence signals are the sole determinant of RNAPIII termination has also been challenged in the fission yeast *Schizosaccharomyces pombe* by a recent report, showing that one of the homologs of the *S. cerevisiae* Sen1 (hereafter designated as *Sp* Sen1) is involved in RNAPIII termination in vivo (*31*). Deletion of this gene that, in *S. pombe*, is nonessential leads to a global shift of RNAPIII occupancy downstream of tRNA genes, consistent with the notion that *Sp* Sen1, in addition to T-tracts, is required for RNAPIII termination in this organism. The precise role of *Sp* Sen1 in termination and its mechanism of action were, however, not addressed in this study.

Thus, much uncertainty remains about the relative contribution of sequence elements, RNA structures, and trans-acting factors to the efficiency of RNAPIII transcription termination. In addition, to what extent the different termination mechanisms are evolutionarily conserved remains an open question.

In the present study, we combine high-resolution genome-wide approaches with in vitro transcription termination assays using highly purified components to dissect the mechanism of RNAPIII transcription termination in *S. cerevisiae*. We observe that termination at the primary terminator of RNAPIII-dependent genes (i.e., the first T-tract after the gene) is only partially efficient and, thus, a considerable fraction of polymerases terminate in the downstream region. We provide in vivo and in vitro evidence that the helicase Sen1 plays a global role in RNAPIII transcription termination and that this function relies on the interaction of its N-terminal domain (NTD) with RNAPIII. However, we find that Sen1 contributes very little to the efficiency of primary termination and that it mainly functions as a fail-safe mechanism to promote the termination of RNAPIIIs that override the first termination signal. Our data indicate that only T-tracts within a particular length range are sufficient to promote autonomous termination by RNAPIII. Nevertheless, we show that tRNA genes often contain suboptimal termination signals and that their capacity to induce termination can be complemented by Sen1 and by secondary structures of the nascent RNA. These two factors act in a mutually exclusive manner since the presence of RNA structures prevents the loading of Sen1 onto the transcript, which is strictly required for Sen1-mediated termination. While Sen1 can also promote the release of RNAPIII at pausing sites other than T-tracts, we find that RNA structures can only function in association with canonical termination signals. Together, our data allow revisiting former models for RNAPIII transcription termination and offer a novel and detailed view of how intrinsic components of the EC (i.e., T-tracts and RNA structures) and the extrinsic factor Sen1 concur to promote efficient termination of RNAPIII transcription.

## RESULTS

### The NTD of Sen1 interacts with RNAPIII

**Fig. 1.**
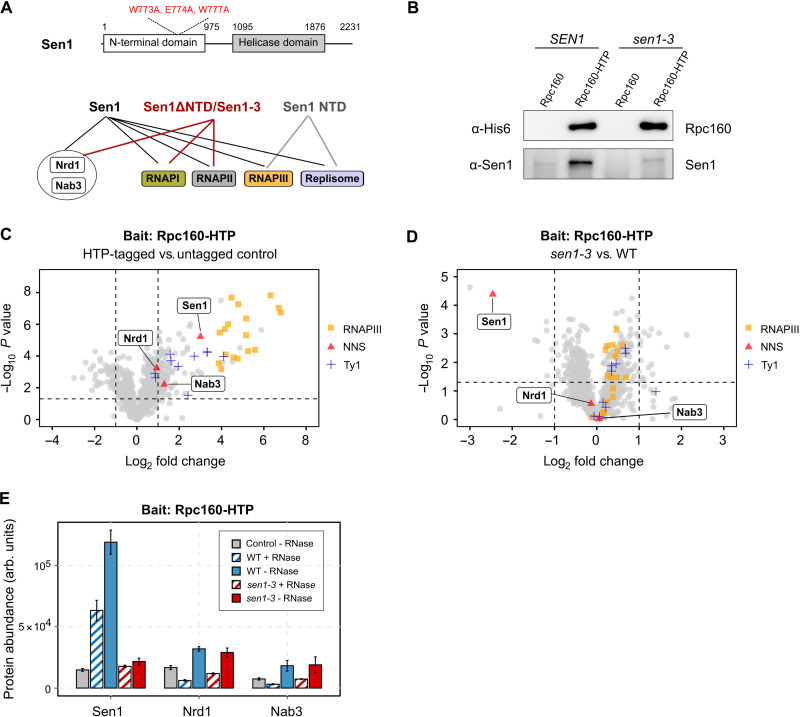
The NTD of Sen1 interacts with RNAPIII. (**A**) Summary of the results of co-IP–MS experiments using different versions of TAP-tagged Sen1 as baits that are included in Table 1. A scheme of Sen1 protein indicating the different functional domains and the position of the mutations introduced in the *sen1-3* strain is shown on the top. Globular domains are denoted by solid bars, while protein regions predicted to be intrinsically disordered are indicated by a line. (**B**) Western blot analysis of a representative co-IP experiment using a C-terminally His_6_–TEV–Protein A (HTP)–tagged version of the largest subunit of RNAPIII (Rpc160) as the bait. (**C** and **D**) Label-free quantitative MS analysis of co-IP assays using Rpc160-HTP as the bait. Data correspond to experiments performed in the absence of RNase A treatment. (C) Volcano plot representing the enrichment of the detected proteins in the HTP-tagged strain relative to the untagged control in a WT (*SEN1*) background. (D) Quantitative comparison of the proteins that are associated with tagged RNAPIII in a *sen1-3* mutant relative to the WT. Only proteins with a fold change of ≥2 relative to the control and *P* < 0.05 is considered as significantly changed among the compared conditions. (**E**) Comparison of the abundance (arb. units: arbitrary units) of the different NNS components in Rpc160-HTP coimmunoprecipitates with or without treatment with RNase A.

*S. cerevisiae* Sen1 is a modular protein composed of a large NTD (amino acids 1 to 975), a central helicase domain (amino acids 1095 to 1867), and a C-terminal disordered region (amino acids 1930 to 2231; see Fig. 1A). We have recently shown that the NTD is essential for viability and for the termination of RNAPII transcription and that it recognizes the C-terminal domain (CTD) of RNAPII, although it is not the only RNAPII-interacting region in Sen1 (*32*). In a quest for other functional interactions mediated by the Sen1 NTD, we performed coimmunoprecipitation (co-IP) experiments, followed by mass spectrometry (MS) analyses using either a full-length or a ΔNTD version of Sen1 as a bait (Table 1 and table S1). We expressed both *SEN1* variants from the *GAL1* promoter (p*GAL1*) because only overexpression of the *sen1*Δ*NTD* allele supports viability (*32*). In agreement with previous reports (*33*, *34*), we detected a ribonuclease (RNase)–resistant interaction of Sen1 with its partners within the NNS complex’s Nrd1 and Nab3, several replication- and transcription-related factors, and with the three RNAPs. Notably, deletion of the NTD abolished the association of Sen1 with RNAPIII and most replication factors without markedly affecting other interactions. Additional co-IP/MS experiments using the isolated NTD as a bait confirmed the interaction with replication factors (e.g., Ctf4) and RNAPIII subunits, strongly suggesting direct protein-protein interactions between the NTD and these factors (Table 1 and table S2).

**Table 1.** MS analyses of co-IP experiments using different versions of Sen1 as bait. Values correspond to the mascot score. Baits are fused to a tandem affinity purification (TAP) tag. ND, not detected. Note that the mascot score depends on the size of the protein, and therefore, truncated versions of Sen1 have lower values. The full datasets and the results of additional replicates are included in tables S1 to S3.

		**TAP-Sen1 versus TAP-Sen1ΔNTD**			**TAP-NTD**		**Sen1-TAP versus Sen1-3–TAP**		
**Protein**	**Complex**	**Ctrl**	**Sen1**	**Sen1ΔNTD**	**Ctrl**	**Sen1 NTD**	**Ctrl**	**Sen1**	**Sen1-3**
Sen1	NNS	50	24,819	14,182	153	4749	0	8280	7299
Nrd1	NNS	0	439	665	ND	ND	0	315	240
Nab3	NNS	0	417	575	ND	ND	0	185	89
Ctf4	Replisome	0	2465	0	19	1343	0	181	0
Mrc1	Replisome	0	40	0	ND	ND	0	63	21
Rpa190	RNAPI	126	463	1326	31	0	492	2041	1812
Rpa135	RNAPI	87	122	664	29	0	400	1505	1060
Rpb1	RNAPII	0	2770	3003	81	102	113	2313	2494
Rpb2	RNAPII	0	2302	2328	86	55	34	1482	1684
Rpc160	RNAPIII	0	7479	0	0	358	25	2216	59
Rpc128	RNAPIII	0	4731	0	0	125	42	938	204
Rpc82	RNAPIII	0	2735	0	0	233	0	1135	34
Rpc53	RNAPIII	68	1255	183	0	56	0	500	34
Rpc37	RNAPIII	0	1982	0	0	132	0	197	50
Rpc34	RNAPIII	0	2022	0	0	28	0	433	174
Rpc31	RNAPIII	0	1212	0	0	19	0	426	0
Rpc25	RNAPIII	0	410	0	0	0	0	79	0
Rpc17	RNAPIII	0	422	0	0	91	0	128	0
Rpc11	RNAPIII	0	191	0	15	37	33	138	62

The interaction of the NTD of Sen1 with the replisome was found to depend on the replication factors Ctf4 and Mrc1 in a parallel, collaborative study (*33*). In that work, we found that a combination of three point mutations in a conserved region of the Sen1 NTD (W773A, E774A, and W777A; defining the Sen1-3 variant) abolishes the interaction with these proteins. We showed that Sen1-3 is expressed at similar levels as wild-type (WT) Sen1 and is fully proficient for terminating the transcription of NNS target genes (*33*). To assess whether these mutations also affect the association with RNAPIII, we analyzed the protein interactome of Sen1-3 by co-IP/MS (Fig. 1A, Table 1, and table S3). The interaction with RNAPII was not substantially altered in this mutant, in agreement with its proficiency in RNAPII transcription termination (*33*). We observed that the mutations introduced in Sen1-3 strongly affect the interaction with RNAPIII subunits.

These results are compatible with the notion that the same surface of Sen1 mediates mutually exclusive interactions with the replisome and RNAPIII. Alternatively, the interaction between Sen1 NTD and RNAPIII could be mediated by the replisome. To distinguish between these possibilities, we conducted quantitative MS and Western blot analyses on RNAPIII coimmunoprecipitates from WT and *sen1-3* cells (Fig. 1, B to D, and table S4). We observed a clear association of RNAPIII with protein components of the Ty1 transposon, which was previously reported and validates our experimental conditions [Fig. 1C (*35*)]. While Sen1 was among the most enriched RNAPIII interactors, we did not detect the two replisome-anchoring factors, Ctf4 and Mrc1, indicating that Sen1 interacts in a mutually exclusive manner with RNAPIII and the replisome. RNase A treatment induced a ~2-fold decrease in the level of RNAPIII-bound Sen1, indicating that this interaction is also partially mediated or stabilized by the RNA (Fig. 1E). As expected, the association of Sen1-3 with RNAPIII was strongly reduced compared to WT Sen1 (Fig. 1D), even in the absence of RNase treatment, suggesting that the protein-protein interaction mediated by Sen1 NTD is a major prerequisite for the association of Sen1 with RNAPIII transcripts. Notably, the Sen1 NNS partners Nrd1 and Nab3 were very poorly enriched in RNAPIII coimmunoprecipitates (Fig. 1, C to E), strongly suggesting that Sen1 plays a role in RNAPIII transcription termination independently from its function within the NNS complex. Together, our results support the notion that Sen1 associates with RNAPIII and the replisome within two alternative complexes that are also distinct from the NNS complex and likely exert different functions.

### Sen1 is required for efficient termination of RNAPIII transcription in vivo

**Fig. 2.**
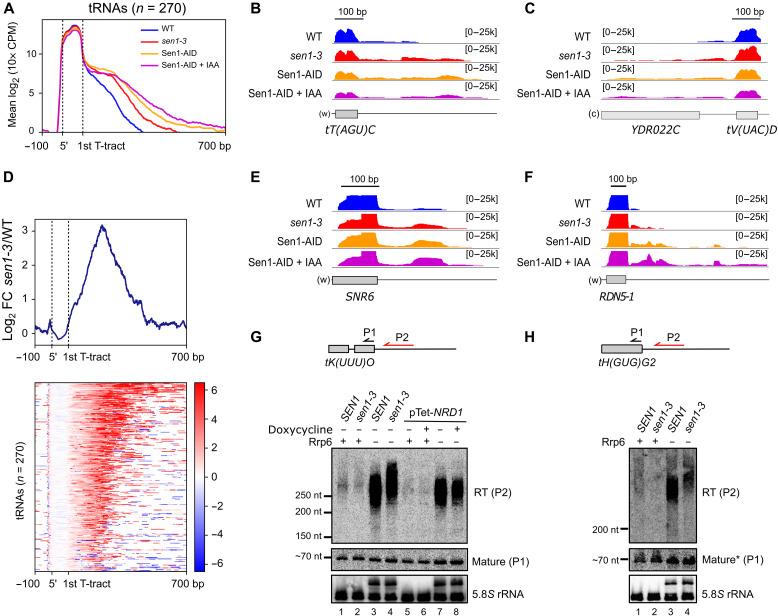
The interaction of Sen1 with RNAPIII is globally required for efficient transcription termination at RNAPIII-dependent genes. (**A**) Metagene analysis of the RNAPIII distribution around tRNA genes. The signal covering the region between the 5′ and the primary terminator is scaled to 100 bp. Sen1-AID, strain expressing an AID version of Sen1. (**B**, **C**, **E**, and **F**) Integrative Genomics Viewer (IGV) screenshots of individual genes displaying termination defects upon mutation or depletion of Sen1. “w” and “c,” Watson and Crick strands, respectively. Values under brackets correspond to the signal scale expressed in 10× counts per million (CPM). (**D**) Heatmap analysis representing the log_2_ of the fold change (FC) of the RNAPIII signal around tRNA genes in *sen1-3* relative to the WT. Top: Summary plot of the mean values. (**G** and **H**) Northern blot analysis of transcripts derived from two tRNA genes in the indicated backgrounds. The approximate position of the probes (P1 and P2) used for the detection of the different RNA species (RT and mature tRNA) is shown on the top. RNA and DNA probes are indicated in red and black, respectively. pTet-NRD1, strain/s expressing *NRD1* from a Tet-Off promoter. Nrd1 was depleted by incubation with doxycycline for 10.5 hours. The 5.8*S* rRNA is used as a loading control. 5.8*S* precursors are detected in the Δ*rrp6* background because Rrp6 processes the 5.8*S*. An asterisk indicates that the signal of mature tH(GUG)G2 corresponds to the same samples loaded in the blot in (G).

The most widely accepted model for RNAPIII transcription termination posits that the polymerases recognize a cis-acting element composed of a stretch of Ts on the nontemplate DNA and is released without the need for additional trans- or cis-acting factors [reviewed in (*2*, *23*)]. However, the evidence supporting a direct interaction between RNAPIII and Sen1 prompted us to investigate a possible role for the latter in terminating RNAPIII transcription. To this end, we generated high-resolution maps of transcribing RNAPIII by CRAC (cross-linking analysis of cDNAs) (*36*, *37*). Briefly, the nascent RNAs are cross-linked to RNAPIIIs in vivo using ultraviolet (UV) light, and the RNAPIII-RNA complexes are purified under stringent conditions. The extracted RNAs are then used to generate cDNAs that are deep-sequenced, providing the position of RNAPIIIs with nucleotide resolution. We performed these experiments in WT or *sen1-3* cells and in a Sen1–AID (auxin-inducible degron) strain, which allowed assessing the effect of Sen1 depletion (Fig. 2).

We obtained very specific and reproducible RNAPIII occupancy signals in the cross-linked samples relative to uncross-linked controls, with most reads mapping at RNAPIII-dependent genes (fig. S1, A to C). Consistent with a former genome-wide study (*38*), our metagene analyses revealed significant RNAPIII signals downstream of the first T-tract after the 3′ end of tRNA genes (hereafter referred to as the primary terminator), indicating that termination at this sequence element is only partially efficient in vivo (Fig. 2, A to C). We observed a clear increase in the RNAPIII signal downstream of the primary terminator in the *sen1-3* mutant, indicating that the interaction with Sen1 promotes termination of RNAPIII, either at the primary terminator or downstream of it. Read-through (RT) transcription was also increased in the Sen1-AID strain even under nondepletion conditions, most likely because the presence of the tag affects the amount or the function of Sen1 even in the absence of auxin, as observed for other proteins. Transcriptional RT was further exacerbated when Sen1 was depleted by the addition of the auxin analog indole-3-acetic acid (IAA). The stronger effect of Sen1 depletion relative to the *sen1-3* mutation might imply either that Sen1-3 can still interact weakly with RNAPIII in vivo or that Sen1 functions in RNAPIII termination to some extent in the absence of interaction with the polymerase. Nevertheless, because full depletion of Sen1 also affects the termination of many RNAPII noncoding RNA genes, we focused on the more specific *sen1-3* mutant for the rest of our study.

Heatmap analyses of the RNAPIII differential signal (log_2_ ratio) in the *sen1-3* mutant relative to the WT showed that an increase in the signal downstream of the primary terminator could be observed for the vast majority of tRNA genes (Fig. 2D). Furthermore, inspection of other RNAPIII-dependent genes such as the 5*S* and U6 genes revealed similar transcription termination defects, indicating that the role of Sen1 in favoring RNAPIII transcription termination is not restricted to tRNA genes (Fig. 2, E and F). Together, our results indicate that Sen1 is globally required for a fully efficient termination of RNAPIII transcription in vivo and that this Sen1 function relies, to a large extent, on its interaction with RNAPIII.

### Sen1 functions in RNAPIII transcription independently of the NNS complex

**Fig. 3.**
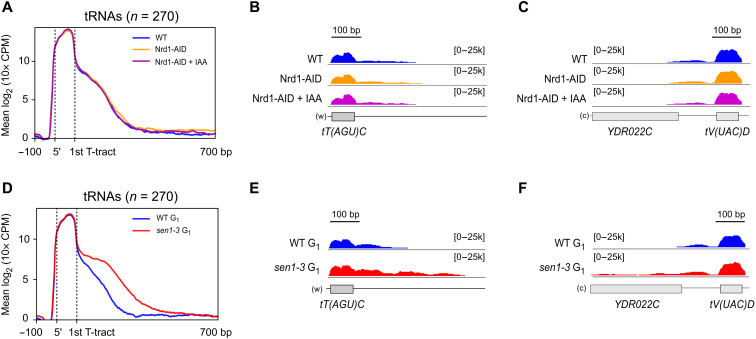
The function of Sen1 in RNAPIII transcription termination does not rely on Sen1 interaction with its partners Nrd1 and Nab3 or with the replisome. (**A**) Metagene analysis of the RNAPIII distribution around tRNA genes as in Fig. 2 in a WT or in a Nrd1 AID strain in the absence or in the presence of IAA. Additional experiments validating the efficiency of Nrd1 depletion on well-characterized NNS target RNAs are included in fig. S2. (**B** and **C**) Individual examples of tRNA genes that exhibit clear termination defects in *sen1-3* (see Fig. 2, B and C) but not under Nrd1-depleted conditions. (**D**) Metagene analysis as in Fig. 2A but in cells blocked in the G_1_ phase of the cell cycle. (**E** and **F**) Individual examples of tRNA genes that display termination defects in the *sen1-3* mutant in cells blocked in G_1_ and in asynchronous cells (compare with Fig. 2, B and C).

Nrd1 and Nab3 have been found to bind the precursors of several tRNAs in vivo (*18*), and it remains possible that these proteins also partake in RNAPIII termination, although they did not appear significantly associated with RNAPIII in our MS analyses (Fig. 1E). To address this possibility, we conducted RNAPIII CRAC experiments in a Nrd1-AID strain. Depletion of Nrd1 upon treatment with IAA for 1 hour was sufficiently efficient to provoke clear termination defects at two well-characterized noncoding genes transcribed by RNAPII (i.e., *NEL025c* and *SNR13*; see fig. S2C). However, neither the metagene analyses of RNAPIII distribution around tRNAs (Fig. 3A) nor the inspection of individual RNAPIII-dependent genes (Fig. 3, B and C) revealed any significant effect on RNAPIII transcription termination efficiency. We conclude that, unlike Sen1, Nrd1 is not required for efficient termination of RNAPIII transcription. Because Nab3 is not known to function separately from Nrd1, our results indicate that Sen1 plays a role in RNAPIII transcription independently from the NNS complex.

### The function of Sen1 in RNAPIII transcription termination is not mediated by the replisome

Our analyses of Sen1 and RNAPIII protein interaction network support a model whereby Sen1 interacts with RNAPIII and the replisome in a mutually exclusive manner. However, they do not exclude the possibility that the replisome mediates the loading of Sen1 onto RNAPIII, for instance, when a collision between these complexes occurs (e.g., Sen1 could interact sequentially with the replisome and RNAPIII). RNAPIII transcription units are indeed hotspots of conflicts between the transcription and the replication machineries (*39*). Therefore, we considered the possibility that Sen1 might only function in RNAPIII transcription termination in the presence of ongoing replication. To explore this possibility, we performed parallel RNAPIII CRAC experiments in asynchronous cells and in cells arrested in the G_1_ phase by treatment with α factor, in a WT and a *sen1-3* background (G_1_ arrest was verified by florescence-activated cell sorting analysis; fig. S1, D and E). We observed a very similar RNAPIII pattern in G_1_-arrested and asynchronously growing cells (Figs. 2, A to C, and 3, D and E), namely prominent RNAPIII termination defects in *sen1-3*. The finding that abolishing the interaction between Sen1 and RNAPIII reduces the efficiency of termination even in the absence of the replisome (i.e., G_1_-arrested cells) indicates that Sen1 plays a role in the termination of RNAPIII transcription independently of its association with the replisome.

### Sen1 operates in a fail-safe transcription termination pathway

Our genome-wide data indicate that the association of Sen1 with RNAPIII globally increases the efficiency of transcription termination. However, these results are consistent with both a function for Sen1 in promoting termination at the primary terminator and a role in removing polymerases that constitutively escape primary termination.

To distinguish between these possibilities, we first analyzed the distribution of the RNAPIII CRAC signal in WT and *sen1-3* cells. The total transcription levels, inferred from the RNAPIII signal within the gene body, were virtually identical in WT and *sen1-3* cells, indicating that the mutations in Sen1-3 do not affect transcription initiation or elongation (fig. S3, A and B).

**Fig. 4.**
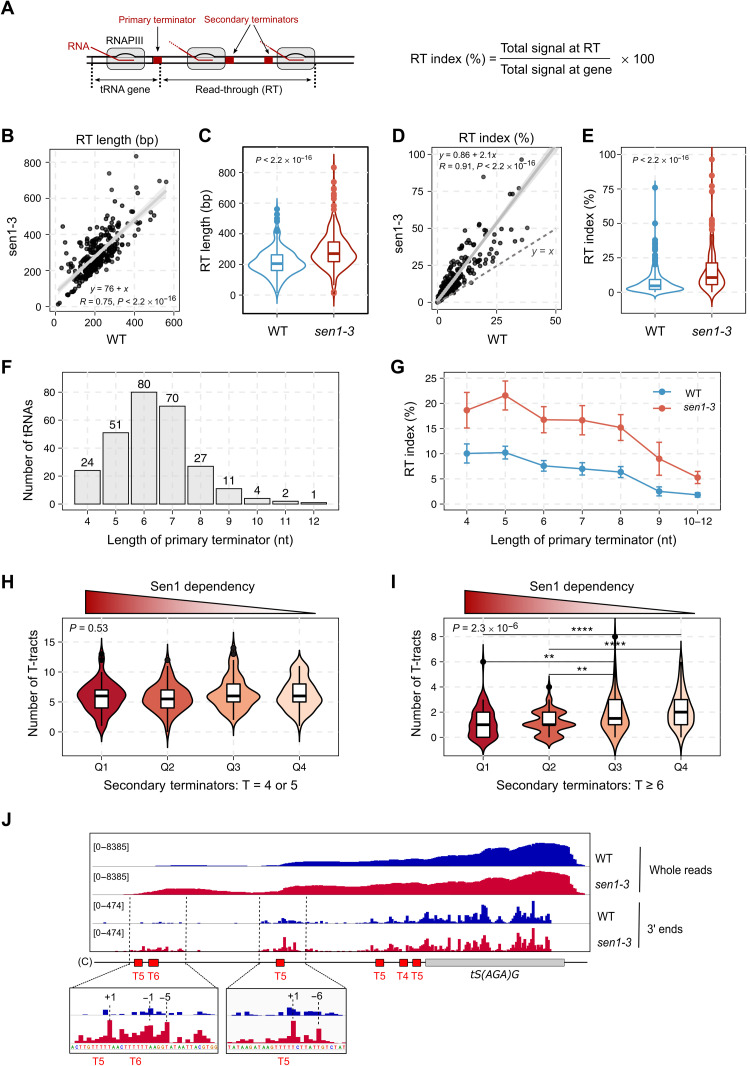
Sen1 functions mainly on secondary termination. (**A**) Scheme of tRNA genes and parameters used to estimate the transcription termination efficiency. (**B** and **C**) Comparison of the RT length for the different tRNA genes in *sen1-3* relative to the WT. (B) Correlation plot with the confidence interval in gray. *R*, Pearson’s correlation coefficient; *P*, Pearson’s correlation *P* value. (C) Violin plot showing the distribution of RT lengths in the WT and in *sen1-3*. *P*, *P* value calculated with the Wilcoxon test. (**D** and **E**) Comparison of the RT index measured in the indicated strains for each tRNA gene as in (B) and (C). Three outliers in *sen1-3* are excluded. (**F**) Histogram representing the number of tRNA genes that have a primary terminator of each indicated length. Only consecutive Ts are considered as part of primary terminators. (**G**) Analysis of the RT index of tRNA genes grouped according to the length of their primary terminator. Data points are the average values. Error bars denote the standard error. (**H** and **I**) Analysis of the number of “weak” (H) or “strong” (I) terminators within the 700-bp region downstream of the primary terminator of tRNA genes. Quartiles (Q) were defined by the tRNA gene ranking obtained in the heatmap analyses in Fig. 2D, where Q1 includes 25% of the genes with the highest impairment in transcription termination in *sen1-3*. *P*, *P* value for the comparison of the four groups (Kruskal-Wallis test). *P* values of pairwise comparisons, **P ≤ 0.01 and ****P ≤ 0.0001. (**J**) IGV screenshot of a tRNA gene showing the distribution of RNAPIII signal in the WT and *sen1-3*. Insets are zoom-in views of the main regions where RNAPIII accumulates in *sen1-3*. Coordinates in insets correspond to the position relative to the beginning of the nearest downstream T-tract.

We then computed for each tRNA gene both the RT index (i.e., the ratio of the RNAPIII signal downstream versus upstream of the primary terminator) and the RT length (i.e., the distance between the primary terminator and the 3′ end of the RT signal) in the WT and in *sen1-3* (Fig. 4, A to E). For most genes, we observed an increase in the RT index in *sen1-3* cells compared to WT cells (Fig. 4, D and E), which is compatible with Sen1 functioning in primary or in secondary termination, since failure in either one of these processes alone would result in the accumulation of RNAPIIIs within RT regions. Computing the total RNAPIII signal in the RT region instead of the RT index provided very similar results (fig. S3, C and D), as expected because the overall signal in the gene body is unchanged in the *sen1*-3 mutant. Thus, comparing the RT indexes is equivalent to comparing the signals in the unique RT regions, which avoids possible issues related to the determination of the signals at repeated tRNA sequences (see Materials and Methods for details).

However, the heatmap analyses shown in Fig. 2D revealed that for most tRNA genes, very little or no RNAPIII accumulation could be observed immediately after the primary terminator in the mutant, with the largest increase in RNAPIII signal occurring further downstream, arguing against a major role for Sen1 at the primary termination site. Consistent with this notion, we observed a clear increase in the RT length in the mutant (Fig. 4, B and C), indicating that polymerases that have escaped primary termination transcribe for longer because downstream termination is defective in the Sen1 mutant.

Because termination defects would lead to the production of different RNA species from tRNA genes depending on whether they occur at the primary terminator or at RT regions, we set out to analyze these RNAs by Northern blot (Fig. 2, G and H). Mature tRNAs are generated by termination at the primary terminator and eventually by the processing of short 5′ and 3′ extensions. Therefore, defects in primary termination are expected to result in lower amounts of mature tRNAs with a concomitant increase in the amount of RT transcripts. We could only detect RT RNAs for the tRNA genes *tK(UUU)O* and *tH(GUG)G2* in the absence of the exosome-associated exonuclease Rrp6 (Fig. 2, G and H), consistent with former data indicating that RT species are degraded by the RNA exosome (*38*). In the case of *tG(GCC)F2*, simultaneous deletion of *RRP6* and depletion of the tRNase Z endonuclease Trz1, involved in the processing of tRNA precursors (*40*), were required for the strong detection of RT transcripts (fig. S2A), indicating that RT transcripts can also be targeted by Trz1.

In all these cases, we did not observe a significant decrease in the abundance of mature tRNAs in *sen1-3*, not even upon depletion of Trz1, excluding the possibility that RT transcripts are recognized as tRNA precursors by this endonuclease and cleaved to generate mature tRNAs (fig. S2, A and E). Thus, unlike tRNA short 3′ end extensions, in the case of RT transcripts produced in either a WT or a mutant context, Trz1-mediated cleavage results in full degradation. The overall abundance of RT RNAs was similar in the WT and in *sen1-*3, but these species were globally longer in *sen1-3* cells, confirming CRAC data suggesting that they result from defective Sen1-dependent termination occurring downstream of tRNA primary terminators (Fig. 2, G and H, and fig. S2A). This increase in size was not observed, as expected, when the NNS subunit Nrd1 was depleted, consistent with the Nrd1-AID RNAPIII CRAC data (Figs. 2G and 3, A to C, and fig. S2D).

To further support the notion that Sen1 functions mainly on RNAPIIIs that have escaped the primary termination site, we performed more detailed analyses of our CRAC data. If Sen1 does not function in primary termination, then its failure to interact with RNAPIII should affect similarly genes with weak or strong primary terminators. On the basis of in vitro data, the minimal length for a functional terminator is five Ts (*24*, *26*), but six Ts are required for relatively efficient termination, and it is generally assumed that the termination efficiency is higher as the T-tract length increases, although the sequences flanking the T-tract have also been shown to affect the strength of the terminator (*41*). In partial agreement with these notions, we observed that (i) the first T-tract rarely contains four Ts, (ii) six Ts and seven Ts are the most frequent terminators at this position, and (iii) tracts longer than eight Ts are rarely found as primary terminators (Fig. 4F). We analyzed the RT index of tRNAs clustered according to the length of their primary terminator, and, as expected, we found that the RT index in these clusters tends to decrease as the T-tract length increases (Fig. 4G) in inverse correlation with the termination efficiency. A similar trend was observed when computing the total RNAPIII signal at the RT region instead of the RT index (fig. S3E). In *sen1-3* cells, the RT index increases similarly for all clusters, suggesting that having an inefficient primary terminator does not make termination more sensitive to Sen1, arguing against a role of Sen1 at these sites.

The region downstream of tRNA genes contains T-stretches that were previously proposed to play a role as secondary termination sites (*38*). We considered the possibility that Sen1 might be preferentially required for tRNA genes having a lower number of secondary termination sites or less efficient ones. To address this possibility, we ranked the different tRNAs according to the extent of the RNAPIII accumulation in *sen1-3* relative to WT cells, thus defining a hierarchy of Sen1 dependency. For each tRNA, we computed the number of weak (4 or 5 Ts) or strong (≥6 Ts) terminators in regions of secondary termination, and we compared the average number of terminators of each kind in the different quartiles. We found that the tRNA genes that are more dependent on Sen1 for termination (i.e., Q1) tend to have a lower number of efficient terminators compared to those that are less dependent (i.e., Q3 and Q4). In contrast, the number of weak terminators, which have a lower impact on RNAPIII progression, was similar in all groups of tRNAs (Fig. 4, H and I, and fig. S3, F and G).

These results strongly suggest that Sen1 compensates for the lack of efficient terminators in regions of secondary termination. This could imply that Sen1 improves termination at weak terminators or that it promotes termination at other sequences. A careful analysis of the RNAPIII CRAC signal at individual tRNA genes provided evidence supporting both possibilities (Fig. 4J and fig. S4). Mapping only the 3′ end of the nascent RNA allows obtaining a precise readout of RNAPIII position with single-nucleotide resolution. We observed a very little, if any, effect of the *sen1-3* mutation at positions around the primary terminator, while RNAPIII was clearly found to accumulate preferentially not only around T-tracts but also at other sequences, in the downstream regions. Together, our results support the notion that Sen1 does not play a prominent role in primary termination and rather promotes the release of RNAPIIIs that pause within regions of secondary termination.

### Sen1 can promote termination of RNAPIII transcription in vitro

**Fig. 5.**
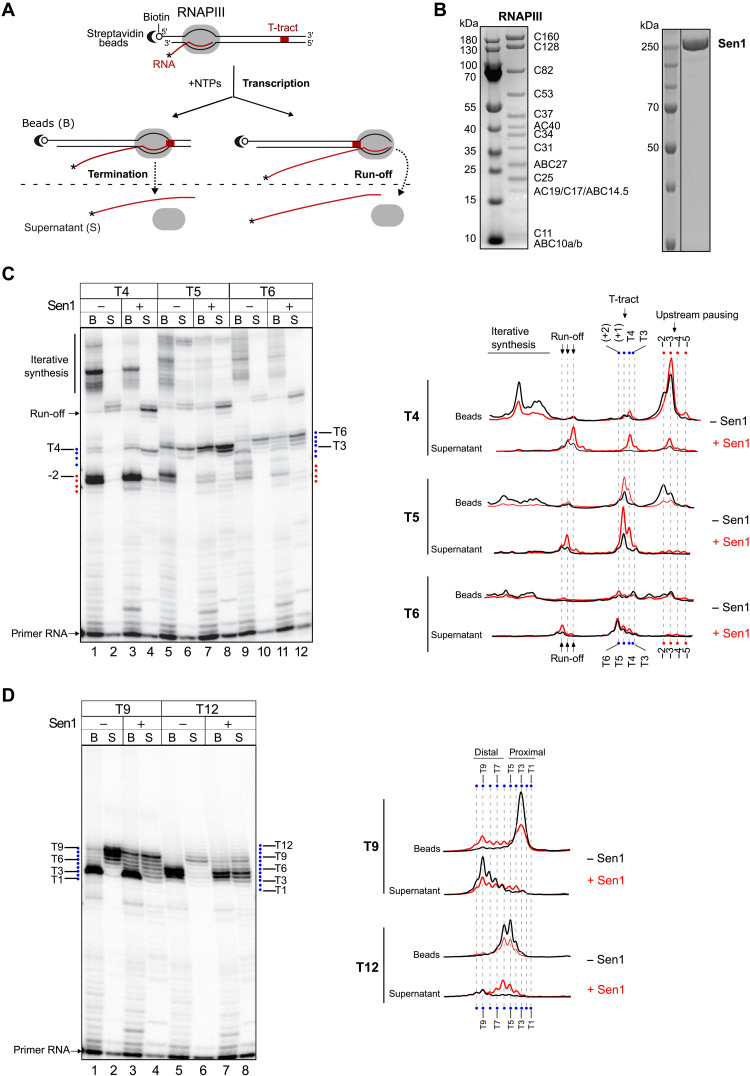
Sen1 can induce termination of RNAPIII transcription in vitro. (**A**) Scheme of an in vitro transcription termination assay. Ternary ECs composed of purified RNAPIII, nascent RNA, and DNA transcription templates are assembled by stepwise addition of the different components (see Materials and Methods) and associated with streptavidin beads via the 5′ biotin of the nontemplate strand to allow subsequent separation of bead-associated (B) and supernatant (S) fractions. An asterisk denotes the radioactive label at the RNA 5′ end to allow its detection. Each transcription template contains a T-tract of a particular length on the nontemplate strand. After addition of nucleotides, RNAPIII transcribes and pauses at different positions. RNAPIIIs that pause at T-tracts can undergo transcription termination and be released to the supernatant, remain paused, and thus associated with the beads, or resume transcription. Polymerases that reach the end of the template can either run off or perform iterative synthesis. Comparison of the amount of transcripts in the beads and the supernatant fractions provides an estimate of the efficiency of termination. (**B**) SDS–polyacrylamide gel electrophoresis (PAGE) analyses of the protein preparations used in in vitro transcription termination assays. (**C** and **D**) Analyses performed on templates containing T-tracts composed of 4 (T4), 5 (T5), 6 (T6), 9 (T9), or 12 (T12) consecutive Ts. Left: Denaturing PAGE analysis of transcripts from one of three independent in vitro transcription termination assays. Right: Profile of the signal over the region of interest for each gel lane. The position of the nucleotides of interest was determined by migrating in parallel a radioactively labeled ladder.

We have previously demonstrated that Sen1 can directly promote the termination of RNAPII transcription in a sequence-independent manner (*14*). To assess whether Sen1 can also directly induce RNAPIII transcription termination and whether it requires the presence of canonical termination signals, we used an in vitro transcription termination system containing purified proteins (i.e., RNAPIII and full-length Sen1), transcription templates, and nascent RNA (Fig. 5, A and B).

We first analyzed the capacity of canonical terminator sequences to induce RNAPIII transcription termination by comparing the behavior of RNAPIII on transcription templates containing T-tracts of variable lengths (i.e., from 4 to 12 Ts; see Fig. 5, C and D, and fig. S5). Consistent with former data (*24*, *26*), we observed only very weak polymerase pausing at the T4 terminator and no detectable RNAPIII release. The T5 terminator induced stronger pausing and intermediate levels of RNAPIII release, while the T6 terminator promoted a very efficient release. Stretches of 9 or 12 Ts induced very strong RNAPIII pausing as virtually no transcription signal could be detected downstream of these terminators, but a substantial proportion of RNAPIIIs remained associated with the proximal part of these long T-tracts (~50% for the T9 and ~80% for the T12). This might be due to the recognition of the distal portion of the T-tract in the downstream DNA by RNAPIII, which might induce strong pausing and disfavor release (see Discussion). For shorter T-tracts, we also observed several prominent pausing sites a few nucleotides upstream of these sequences, both in vitro (Fig. 5C) and in vivo (Fig. 4J and fig. S4), supporting the idea that RNAPIII can sense downstream untranscribed T-tracts.

The presence of Sen1 in the reaction provoked a substantial increase in the levels of transcription termination at the T4 terminator and, to a lesser extent, at the T5 terminator, while no significant effect was observed for the more efficient T6 terminator. This result indicates that Sen1 can enhance RNAPIII release at weak terminators.

We found that Sen1 could also promote the release of RNAPIIIs that are paused at the proximal part of long T-tracts, especially in the case of the T12 terminator, for which roughly 50% of paused RNAPIIIs were released by Sen1. Last, we also observed Sen1-dependent release of RNAPIIIs that are paused at sequences other than T-tracts, for instance, at pausing sites upstream of the canonical terminators, which corroborates our in vivo RNAPIII CRAC analyses (Fig. 4J and fig. S4). Together, these results indicate that Sen1 can both enhance termination at inefficient terminators and promote termination at unrelated sequences.

### Sen1 uses a similar mechanism to terminate transcription of RNAPII and RNAPIII

**Fig. 6.**
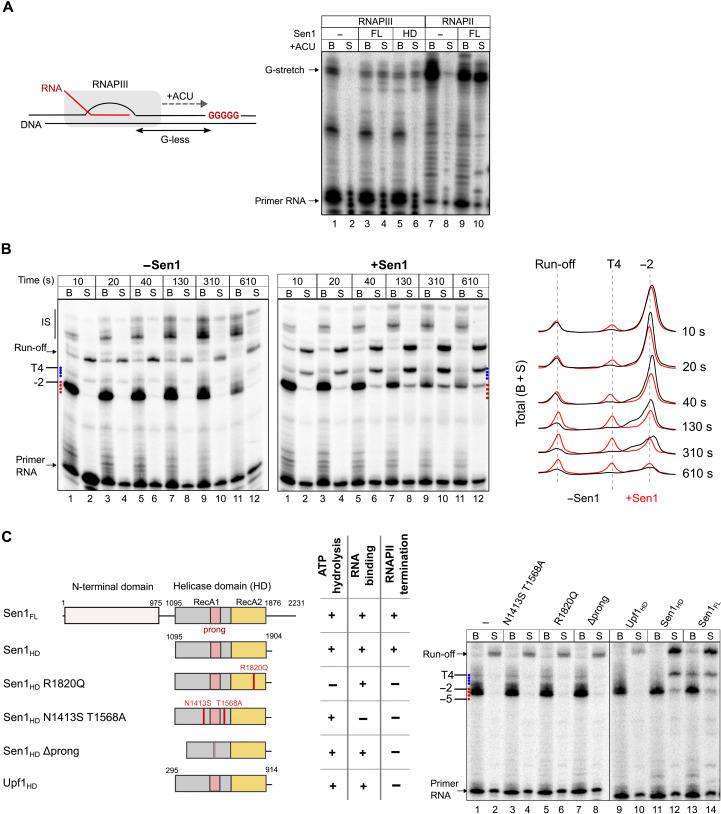
Analysis of the mechanisms of Sen1-mediated termination of RNAPIII transcription. (**A**) Analysis of the capacity of Sen1 to promote the release of RNAPIII paused at a sequence other than a T-tract. The transcription templates contain a G-less cassette, followed by a stretch of Gs to induce RNAPIII stalling at the first G in the absence of GTP. Experiments with purified RNAPII were performed as a positive control for Sen1 activity and provided results similar to our former studies (*19*, *21*). (**B**) Time-course in vitro transcription termination assay performed on transcription templates containing a T4 terminator. All reactions were performed in parallel but migrated on different gels. Left: Representative gel of one of two independent experiments. Right: Profile of the total signal (beads + supernatant) over the region of interest. (**C**) Analysis of the role of different protein regions and activities of Sen1 in RNAPIII transcription termination. Left: Scheme of the different proteins used in in vitro transcription termination assays and summary of the relevant phenotypes. The different variants of Sen1 helicase domain (HD) were purified and characterized within the frame of a previous study (*21*). A constitutively active version of the Upf1 helicase domain that has helicase activity but cannot induce RNAPII transcription termination in vitro is used as a negative control [see (*14*)]. The Δprong version of Sen1 helicase domain contains a deletion of amino acids 1461 to 1554 corresponding to most of this subdomain. Right: Representative gel of one of two independent experiments. All reactions were performed in parallel but migrated on different gels. IS, iterative synthesis.

According to previous studies, canonical terminators contain signals that induce both RNAPIII pausing and release from the DNA [reviewed in (*2*, *23*)]. The above results indicate that Sen1 requires polymerase pausing but not necessarily the presence of a T-tract for terminating RNAPIII. To further explore this idea, we performed in vitro transcription assays with modified templates containing a G-less cassette, followed by a run of Gs to force the stalling of RNAPIII at the G-stretch in the absence of guanosine 5′-triphosphate (Fig. 6A). Under these conditions, and similarly to what was observed for RNAPII, Sen1 could induce the release of roughly 50% of paused RNAPIIIs, demonstrating that it can terminate transcription at pausing sites other than T-tracts.

We next set out to investigate the mechanism by which Sen1 induces RNAPIII transcription termination. We have previously shown that, to dissociate the RNAPII EC, Sen1 needs to load on the nascent RNA and translocate toward the RNAPII using the energy of ATP hydrolysis. Upon colliding with RNAPII, Sen1 can induce forward translocation of the polymerase, which, in the appropriate context, results in its release from the DNA (*19*). Our in vitro assays support a similar mechanism for RNAPIII release (Fig. 5C), with evidence for “pushing” the EC at sites of pausing. This is, for instance, manifest at the T5 terminator (Fig. 5C, compare lanes 5 and 6 to 7 and 8) where a decrease in the pausing signal at position −2 is not due to release at this position but rather by forward translocation and release at a downstream site. This is best illustrated in a time-course experiment performed with the T4 template, in which we quantified the total signal in the presence and absence of Sen1 (Fig. 6B). If a decrease in the signal from a paused polymerase is due to its release, then the total signal (i.e., beads + supernatant) at that position should not change. On the contrary, if polymerases are “pushed” by Sen1 and eventually released at a later stage, then the signal distribution should be shifted downward. Upon addition of Sen1, we observe such a signal shift and the accumulation of RNA signal over time at positions where Sen1 induces its release. These findings support the notion that Sen1 promotes both RNAPIII translocation and its release from the DNA, similarly to what we previously showed for RNAPII.

To further explore the mechanisms of RNAPIII termination by Sen1, we first assessed whether the interaction of Sen1 with RNAPIII, mediated by its NTD, is required for the actual step of polymerase release. To this end, we first analyzed the capacity of the helicase domain of Sen1 alone to induce termination in vitro (Fig. 6C). We have previously shown that this domain is sufficient for inducing the termination of RNAPII transcription (*19*, *21*). Notably, we found that the helicase domain of Sen1 could induce the termination of RNAPIII transcription in vitro as efficiently as the full-length protein (Fig. 6C), suggesting that the association of Sen1 with RNAPIII via its NTD is not a strict requirement for termination but might rather play a role in the recruitment of Sen1 to RNAPIII in vivo. As a negative control, we assessed a catalytically active version of the closely related helicase Upf1 (*42*), which could not provoke the termination of RNAPIII transcription, indicating that termination is not induced unspecifically by any active helicase but rather requires specific Sen1 activities or features (Fig. 6C). Last, we analyzed several mutant variants of the Sen1 helicase domain that are deficient for RNA binding (N1413S T1568A) or ATP hydrolysis (R1820Q) or a mutant that retains the catalytic activity but lacks the “prong” subdomain (i.e., Δ1461–1554), which is essential for RNAPII transcription termination (*21*). We formerly proposed that the prong enters the RNA exit channel, provoking destabilizing conformational changes in the EC [discussed in (*43*)]. None of these mutants could promote RNAPIII transcription termination in vitro, indicating that Sen1 uses the same structural features and activities to induce transcription termination of RNAPII and RNAPIII.

### RNA structures upstream of T-tracts can promote the release of paused RNAPIIIs

The above results indicate that, akin to the RNAPII system, Sen1-mediated termination of RNAPIII transcription involves Sen1 translocation along the nascent transcript, and our former structural and biochemical data showed that Sen1 can only interact with single-stranded RNA (*14*, *21*). tRNAs are highly structured RNA molecules, and for a vast majority of them (i.e., 251 of 270 tRNAs), the spacer between the 3′ end of the mature tRNA and the primary terminator is at most 7 nucleotides (nt). We envisioned that a possible reason for which Sen1 does not function at sites of primary termination is that its binding to the nascent RNA is hindered by the cotranscriptional formation of stable structures in the vicinity of the primary terminator. Conversely, less structured RNAs in the RT region would allow Sen1 loading and function.

**Fig. 7.**
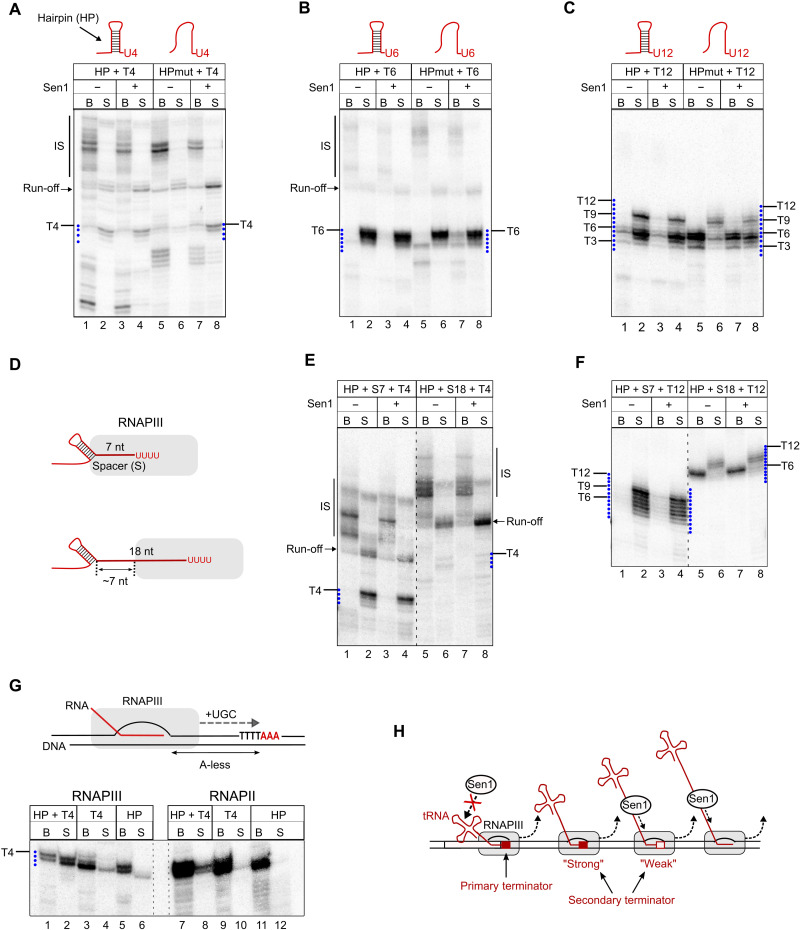
Hairpin-like structures forming in the nascent RNA can complement the function of canonical termination signals. (**A** to **C**) Analysis of the role of RNA structures in transcription termination at a T4 (A), T6 (B), or T12 (C) terminator. In vitro transcription termination assays as in Fig. 5 but with templates introducing a hairpin (HP) in the transcribed RNA immediately upstream of the T-tract. HPmut, template harboring several mutations disrupting hairpin formation (see fig. S4 for details). (**D** to **F**) Analysis of the impact of the positioning of the hairpin relative to the T-tract on its capacity to stimulate RNAPIII release. Experiments performed as in (A) and (C) but with templates containing spacers (S) of the indicated lengths between the hairpin and the T-tract. (**G**) In vitro transcription termination assays performed as in (A) but with templates including an A-less cassette, followed by a stretch of As to induce RNAPIII stalling at the first A in the absence of A. HP, template with the T-tract mutated to CTCT. Experiments were performed also with RNAPII to compare the sensitivity of both RNAPs to termination signals. (**H**) Model for the role of canonical termination signals, RNA structures, and Sen1 in the termination of RNAPIII transcription. At the primary terminator, termination typically involves the action of not only a T-tract but also the structure of the nascent tRNA for T-tracts of suboptimal length. In downstream regions, termination typically occurs either at strong secondary terminators or at weak termination signals with the aid of Sen1, if the nascent RNA is accessible. Sen1 can also promote termination at pausing sites other than T-tracts.

To explore these possibilities, we performed in vitro transcription assays with modified transcription templates containing a natural hairpin from the 5*S* RNA, an RNAPIII-dependent transcript, upstream of T-tracts of different lengths. As a control, we used a mutated hairpin with substitutions in the left arm preventing stem formation (Fig. 7, A to C, and fig. S5). Unexpectedly, the presence of a hairpin in the transcribed RNA could substantially stimulate transcription termination at a T4 terminator, similarly to the addition of Sen1 to the unstructured version of the same RNA (Fig. 7A).

This result indicates that not only Sen1 but also RNA secondary structures can improve the function of weak terminators. In agreement with this idea, the presence of the hairpin did not enhance termination at the T6 terminator, since this sequence already supports full release of paused RNAPIIIs (Fig. 7B). However, the RNA structure could induce the release of polymerases paused at the proximal part of the T12 terminator, even more efficiently than Sen1 (Fig. 7C). These observations support the notion that, similarly to Sen1, RNA hairpins have the capacity to promote RNAPIII release.

Because RNA structures naturally form close to the primary terminator of RNAPIII-dependent genes, we next assessed to what extent secondary structures need to be in proximity to T-stretches to function in termination. To this end, we compared the efficiency of termination on templates containing a T4 or a T12 terminator when the hairpin was located immediately upstream (Fig. 7, A to C) or 7- or 18-nt upstream of the corresponding T-tract (Fig. 7, D to F). We observed a clear enhancement of RNAPIII release at both T4- and T12-containing templates when the hairpin was located immediately upstream or 7 nt away from the T-tract but not in the presence of an 18-nt spacer. These results indicate that RNA structures can enhance transcription termination only when they are in close proximity to T-tracts. The results with the 18-nt spacer provided a tool to address the impact of secondary structures of the RNA on the function of Sen1 in termination. In this case, the formation of a hairpin 18-nt upstream of the T-tract only allows exposing a segment of roughly 7 nt of single-strand RNA outside of the RNAPIII, which is not sufficient for the loading of Sen1 (*21*). Sen1 could not release RNAPIII at the T4 terminator in this construct, likely because of the insufficient single-stranded RNA span between the structure and the polymerase. We observed, however, an increase in the amount of released run-off transcripts in the presence of Sen1, indicating that, at further downstream positions, Sen1 can load on the nascent RNA and promote the release of polymerases at the end of the template (Fig. 7E, lanes 5 to 8).

Together, our results strongly suggest that RNA secondary structures forming in the vicinity of weak primary terminators can markedly improve their function. However, they can also hamper the recruitment of Sen1 to the nascent RNA and thus would likely prevent Sen1 from functioning at primary terminators, regardless of their strength.

### RNA secondary structures can form within the RNA exit channel of RNAPIII

A previously proposed model (*29*) posits that T-tracts are sufficient for RNAPIII pausing but not for its release from the DNA, for which RNA secondary structures would be strictly required. This model opposes the most widely accepted one, which points to an exclusive role for the T-tract in termination. Our data indicate that secondary structures can promote RNAPIII release but only at terminators that, alone, do not support efficient release. We decided to further investigate the mechanism of action of RNA structures in termination. The model of Nielsen *et al.* (*29*) postulates that one of the main functions of T-tracts would be to promote RNAPIII backtracking to bring the polymerase in contact with the nearest upstream structure, which would invade the RNA exit channel of RNAPIII, thus destabilizing the EC. Nonetheless, we have observed that the RNA hairpin is functional when located immediately upstream or very close to a T4 or a T12 sequence, which implies either that (i) RNAPIII transcribes beyond the T-tract to allow the formation of the hairpin, pauses at downstream sequences, and undergoes subsequent backtracking or that (ii) the RNA folds at least partially within the polymerase to induce its release. The former possibility appears to be hardly compatible with the case of the T12 terminator, for which we did not find any evidence of RNAPIII transcribing through the terminator (Fig. 5D).

To address these possibilities, we conducted in vitro transcription assays with modified templates where the RNA hairpin is encoded in an A-less cassette and is followed by a T4 sequence and three As (Fig. 7G and fig. S5). By performing reactions with these templates in the absence of ATP, polymerases cannot transcribe through the terminator and stall at the fourth T of the T4. Under these conditions, the hairpin cannot form outside of the polymerase, with its downstream arm still being within the polymerase.

We observed that stalled RNAPIIIs were released in the presence of the hairpin but not the corresponding mutant version, indicating that transcription through the terminator is not required for the folding of the hairpin, which must occur in the RNA exit channel of RNAPIII. Very little, if any, polymerase release was observed when the T4 sequence was mutated, even in the presence of the hairpin, indicating that the hairpin is not an autonomous termination signal and can only function together with a canonical termination sequence. Last, the concomitant presence of an RNA hairpin and a T-tract induced poor release of RNAPIIs, indicating that these nucleic acid elements function specifically as RNAPIII termination signals.

These findings strongly support the notion that the RNA can fold within the RNAPIII and that backtracking is not required to promote RNAPIII termination. Together, our data support the idea that RNA secondary structures are not absolutely required for RNAPIII termination but can nevertheless function as auxiliary elements that work in concert with weak or defective termination signals.

## DISCUSSION

RNAPIII synthesizes short noncoding RNAs like tRNAs and the 5*S* rRNA that are absolutely essential for mRNA translation and, therefore, for cell growth and survival. Timely termination of RNAPIII transcription is critical not only for the correct synthesis of these RNAs but also for preventing RNAPIIIs from invading neighboring genomic regions and interfering with the function of other machineries that operate in these regions. This is even more relevant considering the high expression levels of RNAPIII-dependent genes.

The traditional model for the termination of RNAPIII transcription posits that termination exclusively relies on the presence of T-tracts at the 3′ end of RNAPIII-dependent genes that are specifically recognized by the polymerase as termination signals. The implication of additional cis-acting factors, such as RNA secondary structures, has been previously proposed (*29*) but has remained hitherto controversial (*30*).

Here, we show that the mechanisms governing RNAPIII transcription termination in vivo are considerably more complex than those represented in former models, involving the interplay between distinct cis-acting elements and the extrinsic termination factor Sen1. We propose an integrated model whereby T-tracts and RNA secondary structures function in concert at primary terminators (and possibly other sites), while Sen1 concurs to release polymerases that have escaped “intrinsic” termination.

### *S. cerevisiae* Sen1 is a fail-safe transcription termination factor for RNAPIII

One of the important findings of this study is the demonstration that Sen1 can directly promote the termination of RNAPIII transcription. Multiple lines of evidence support the notion that Sen1 functions to remove polymerases that have escaped primary termination at the very 3′ end of RNAPIII-dependent genes. These include the high-resolution detection of RNAPIII occupancy by CRAC and the analysis of the different RNA species produced from model tRNA genes (Figs. 2 and 4 and figs. S2 and S4). A mechanistic explanation for our observation that Sen1 cannot operate at primary terminators is provided by our finding that in vitro Sen1 function in termination is hindered by RNA secondary structures, which are typically present in RNAPIII transcripts close to the first terminator.

A recent study has reported that one of the two *S. pombe* homologs of Sen1, *Sp* Sen1, also interacts with RNAPIII and its deletion leads to global defects in RNAPIII transcription termination (*31*). However, some important conclusions of this study are substantially different from the ones supported by our results. It was shown that deletion of *Sp* Sen1 leads to a global downstream shift of the RNAPIII occupancy peak at tRNA genes, as determined by chromatin immunoprecipitation sequencing (ChIP-seq), and a reduction in the levels of mature tRNAs, which we did not observe in *S. cerevisiae*. These findings have been interpreted in support of a model whereby efficient primary termination in *S. pombe* relies on *Sp* Sen1 and would be only partially dependent on intrinsic termination signals. In contrast, in *S. cerevisiae*, primary termination mainly depends on cis-signals (T-tracts and secondary structures), and Sen1 operates in downstream regions to remove RT polymerases. Therefore, in *S. cerevisiae*, Sen1 rather plays an important genome-safeguarding role in preventing the inappropriate extension of RNAPIII transcription.

The divergency between these models might reflect mechanistic differences between the two organisms. For instance, differences in the biochemical properties of the two Sen1 proteins or in the mode that they are loaded onto the nascent transcript might be at stake. Substantial sequence homology between the two proteins can be found only in their helicase domains, and contrary to *S. cerevisiae* Sen1, none of the *S. pombe* Sen1 homologs is essential for viability, interacts with RNAPII, or partakes in RNAPII transcription termination (*31*, *44*). Alternatively, it is possible that the different conclusions of the *S. pombe* study are due to technical differences. CRAC allows assessing the efficiency of termination with single-nucleotide resolution at multiple individual sites for each gene and thus identifying the precise positions where mutation of Sen1 has an impact. In contrast, the ChIP-seq technique, with a lower resolution, does not allow distinguishing a role of *Sp* Sen1 in primary termination from one in the downstream region. Hence, it remains possible that *Sp* Sen1 actually functions similarly to *S. cerevisiae* Sen1. Nevertheless, despite the possible differences, the two studies open up the exciting possibility that this function of Sen1 is conserved in other organisms, including humans.

### The mechanism of Sen1-dependent RNAPIII transcription termination

Although its best-characterized function is the termination of noncoding transcription by RNAPII within the NNS complex, *S. cerevisiae* Sen1 is also implicated in other processes such as the control of R-loop formation, the resolution of transcription-replication conflicts, and DNA repair (*33*, *45*–*47*). The NTD of Sen1 is an important hub for protein-protein interactions that might modulate these different functions of Sen1 (*32*, *33*). The function of Sen1 in RNAPIII termination in vivo strongly depends on the interaction with RNAPIII, which is mediated by a region in the Sen1 NTD containing the amino acids mutated in Sen1-3. This region is not required for termination in vitro, indicating that it is not a critical molecular determinant of the process of RNAPIII release, and therefore, we suggest that it drives the recruitment of Sen1 to RNAPIII, which might be a limiting step for termination in vivo.

Mutation of the same amino acids in Sen1-3 also prevents the interaction with the replisome components Ctf4 and Mrc1 (*33*), yet we show that the interactions of Sen1 with the replisome and RNAPIII are not interdependent but rather mutually exclusive. This suggests either that the same surface mediates the interaction with RNAPIII and the replication fork or that these mutations alter the conformation of two distinct regions of interaction. The observation that Sen1 promotes RNAPIII transcription termination even in the absence of replication (i.e., in G_1_) indicates that the action of Sen1 is not restricted to situations of transcription-replication conflicts. However, we cannot exclude that these conflicts might trigger Sen1-dependent termination in some circumstances.

Our in vitro data strongly support the notion that Sen1 terminates RNAPIII transcription essentially by the same mechanism used to induce RNAPII release, which involves the translocation of Sen1 along the nascent RNA and its collision with the paused polymerase (*14*, *19*, *21*). As we showed for RNAPII, the helicase domain is sufficient for RNAPIII transcription termination, and the essential activities involved in translocation (RNA binding and ATP hydrolysis) and the prong are required. Akin to what we showed for RNAPII, Sen1 “pushes” paused RNAPIII, which either promotes elongation resumption or results in its release from the DNA (Fig. 6). Whether the outcome of pushing (elongation or termination) is determined by alternative, preexisting conformations of paused RNAPIII or it is stochastic remains to be determined.

A former study reported transcription termination defects at RNAPI-dependent genes upon inactivation of Sen1 in vivo (*7*). The interpretation of these data is blurred by the fact that Sen1 inhibition can have multiple indirect effects due to its widespread role in the termination of RNAPII transcription. However, we have found that Sen1 associates with RNAPI in vivo (Table 1) and can also promote the release of paused RNAPI in vitro (fig. S6). Therefore, together, these data could point at a common mechanism of transcription termination operating at the three eukaryotic RNAPs and relying on the helicase Sen1.

### RNA structures are enhancers of canonical termination signals

The two fundamental steps in RNAPIII transcription termination are RNAPIII pausing and release from the DNA. The most widely accepted model (*23*) posits that a stretch of Ts in the nontemplate DNA strand is sufficient for both pausing and release of RNAPIII, whereas an alternative model proposed that RNAPIII release would strictly depend on the presence of an RNA hairpin in the vicinity of the paused RNAPIII (*29*). These fundamental disparities were attributed to differences in the purity of the RNAPIII preparations used in the studies supporting these models (*30*, *48*). Here, we use a high-purity preparation of the RNAPIII holoenzyme validated in structural and functional analyses (*25*, *49*) to investigate the different mechanisms involved in RNAPIII transcription termination.

We find that the capacity of T-tracts to promote RNAPIII pausing is directly linked to the T-tract length, with T4 terminators supporting very little pausing and T ≥ 9 terminators inducing a complete block of RNAPII elongation (Fig. 5, C and D). Our results show that T6 terminators, which are the most frequently found in vivo (Fig. 4F), are not fully efficient in supporting pausing but can induce RNAPIII release in the absence of any adjacent RNA structure (Figs. 5C and 7B), indicating that RNA secondary structures are not always required for termination. In contrast, in the case of T4 terminators, which are essentially nonfunctional in vitro, we find that an adjacent RNA secondary structure can convert these sequences into moderately efficient terminators (Fig. 7A). This behavior likely phenocopies the situation in vivo where the tRNA acceptor stem typically folds very close to the primary terminator and might explain why T4 terminators can be found as primary terminators (Fig. 4F). These sequences remain, however, quite weak terminators, likely explaining why additional T-tracts are most often found in close proximity in vivo (fig. S7).

Notably, in our assays, very long T-tracts (T ≥ 9) are defective in promoting RNAPIII release, and these defects are more pronounced as the length of the T-tract increases. More precisely, we observe that a fraction of RNAPIIIs stall at the proximal portion of these long T-tracts after “reading” only the first 3 to 6 nt of the T-tract and fail to dissociate from the DNA. Our interpretation of these observations is that RNAPIII can recognize the T-tract in the downstream duplex region, either because of its sequence or because of the particular structure T-tracts impose to the DNA helix (*50*). These interactions would stabilize the EC, thus compensating for the destabilizing effect of the weak rU:dA hybrid and the interaction with the unpaired Ts in the nontemplate strand within the transcription bubble. We find that an RNA hairpin forming in the vicinity of these long T-tracts can promote a full release of stalled RNAPIIIs (Fig. 7C), suggesting that in vivo long T-tracts might require the concomitant presence of an adjacent secondary structure to be fully proficient in transcription termination.

We provide mechanistic evidence that RNA hairpins can form within the RNA exit channel of RNAPIII (Fig. 7G). Consistent with this finding, a recent structural study has provided evidence that an RNA hairpin can fold within the RNA exit channel of a bacterial polymerase, leading to a rearrangement of the EC (*51*). Structural comparisons indicate that eukaryotic polymerases can also accommodate these RNA secondary structures within their RNA exit channels (*51*). A very recent structural study on human RNAPI has provided evidence for the presence of double-stranded RNA in the RNA exit channel (*52*). Therefore, we propose that, in the case of RNAPIII, the formation of an RNA hairpin can induce destabilizing conformational changes in RNAPIII that would contribute to the dissociation of the EC but only when a T-tract resides in the polymerase main channel (Fig. 7G). While this work was in progress, a study using a reporter system in human cells provided evidence that an RNA hairpin located close to a short T-tract (T4) can enhance RNAPIII transcription termination in cellulo (*53*), pointing to an evolutionarily conserved role for RNA structures in termination.

Together, our results allow proposing a revisited model for autonomous RNAPIII transcription termination that can partially reconcile former contradictory findings. According to our model, T-tracts are strictly required for termination, but adjacent RNA structures are important auxiliary elements when the length of the T-tract falls outside of the optimal range. Thus, the protein-independent mechanism of termination of RNAPIII transcription has more commonalities with the so-called intrinsic termination pathway for bacterial RNAP than previously appreciated.

### Multiple mechanisms partake in RNAPIII transcription termination

We and others have observed that RNAPIIIs read through the primary terminator quite frequently, and termination at downstream regions was proposed to rely on secondary canonical terminators (*38*). tRNA RT regions contain T-tracts that are more frequent in the sense orientation than in the antisense orientation (fig. S8, A and B), suggesting that they are under positive selection. However, long T-tracts (T > 5) are scarce in these regions (Fig. 4I), suggesting that alternative evolutionary routes have been undertaken for ensuring efficient termination.

We have shown that both RNA structures and the helicase Sen1 can complement the function of short termination signals and that these two factors act in a mutually exclusive manner. Our data indicate that Sen1 would play a more prominent role in termination at RT regions than RNA structures. A possible explanation is that Sen1 can function both at weak terminators and at other pausing sites, while RNA structures can only work when located sufficiently close to a T-tract.

We have observed that the transcripts encoded in the ~250–base pair (bp) region immediately downstream of the primary terminator have a lower propensity to fold into secondary structures than the genomic average (fig. S8, C to E). While this could be partially due to the higher frequency of T-tracts in this region, which lowers the GC content, it might also be a consequence of Sen1 involvement in fail-safe termination. We suggest that “repurposing” the RNAPII transcription termination factor Sen1 for terminating RNAPIII might have a lower evolutionary cost than generating the appropriate arrangements of T-tracts and RNA structures in tRNA RT regions.

These considerations do not exclude the possibility that more than one mechanism operate in secondary termination for the same gene. This is, for instance, illustrated by the *tH(GUG)G2* gene (fig. S2B), where a secondary T8 terminator is present 60-bp downstream of the primary terminator. Termination at this site is independent of Sen1 most likely because a strong secondary structure forms immediately before T8. The fraction of RNAPIIIs that escape termination at this site terminates at downstream sites, in the apparent absence of strong secondary structures, in a Sen1-dependent manner.

Together, our findings reveal the existence of multiple mechanisms cooperating to promote the termination of RNAPIII transcription. We propose that RNA structures contribute to the efficiency of primary termination in some instances, thanks to the natural proximity of the tRNA acceptor stem to the first T-tract, whereas Sen1 would preferentially function at downstream regions (Fig. 7H). Efficient termination is important for the rapid recycling of RNAPIII for new cycles of transcription and thus for maintaining robust expression of tRNAs and other RNAPIII-dependent transcripts that are essential to sustain cell proliferation. In addition, it is crucial to prevent or to minimize the conflicts with other transcribing polymerases and with other DNA-associated machineries.

## MATERIALS AND METHODS

### Construction of yeast strains and plasmids

All the strains used here are listed in table S6. Tagging of *RPC160* with the His_6_–TEV–Protein A (HTP) tag was performed with standard procedures (*54*, *55*) using plasmid pDL599. Plasmid pDL995 for the expression of recombinant Sen1 in insect cells was constructed using the sequence- and ligation-independent cloning method (*56*).

### Coimmunoprecipitation

For immunoprecipitation of proteins expressed under their own promoter, cells were grown on yeast extract, peptone, and dextrose (YPD) medium. For proteins expressed under the control of p*GAL1* (i.e., full-length Sen1, Sen1ΔNTD, and the NTD of Sen1), cells were grown on rich medium containing galactose (20 g/liter) instead of glucose as the carbon source. Cultures (typically 250 ml) were grown to an optical density at 600 nm (OD_600_) of ~1, and then cells were collected by centrifugation and resuspended in 1.5 ml of lysis buffer [10 mM sodium phosphate (pH 7.5), 200 mM sodium acetate, 0.25% NP-40, 2 mM EDTA, 1 mM EGTA, and 5% glycerol] containing protease inhibitors. Suspensions were frozen in liquid nitrogen and lysed using a Retsch MM301 Ball Mill (five cycles of 3 min at 15 Hz). Lysates were clarified by centrifugation at 13 krpm for 30 min at 4°C and, unless otherwise indicated, treated with RNase A (20 μg/ml) for 20 min at 25°C before immunoprecipitation. For HTP-tagged proteins, the extracts were then incubated with 2.5 mg of immunoglobulin G (IgG)–coupled M-280 Tosylactivated Dynabeads (Thermo Fisher Scientific) for 2 hours at 4°C with rotation. After incubation, beads were washed three times with lysis buffer and once with H_2_O and used directly in MS analyses.

For proteins overexpressed from p*GAL1*, IgG Sepharose (GE Healthcare) was used instead. After washes with lysis buffer, beads were washed with TEV cleavage buffer [10 mM tris-HCl (pH 8), 150 mM NaCl, 0.1% NP-40, 0.5 mM EDTA, 1 mM dithiothreitol (DTT), and 5% glycerol], and proteins were then eluted by cleaving the protein A moiety with the TEV protease in TEV cleavage overnight at 4°C.

### MS analysis and label-free quantification

Analysis of Sen1 and RNAPIII coimmunoprecipitates by MS was conducted by the proteomics core facility of the Institut Jacques Monod. Proteins were digested by adding 0.2 μg of trypsin (Promega, Madison, WI, USA) per sample, followed by incubation in 25 mM NH_4_HCO_3_ at 37°C overnight. The resulting peptides were desalted using ZipTip μ-C18 Pipette Tips (Pierce Biotechnology, Rockford, IL, USA) and analyzed using an Orbitrap Fusion equipped with an easy spray ion source and coupled to a nano-LC Proxeon 1200 (Thermo Fisher Scientific, Waltham, MA, USA). Peptides were loaded with an online preconcentration method and separated by chromatography using a Pepmap-RSLC C18 column (diameter, 0.075 mm; length, 750 mm; 750 mm, 2 μm, 100 Å) from Thermo Fisher Scientific, equilibrated at 50°C and operating at a flow rate of 300 nl/min. Peptides were eluted by a gradient of solvent A [H_2_O and 0.1% formic acid (FA)] and solvent B (acetonitrile/H_2_O 80/20 and 0.1% FA); the column was first equilibrated for 5 min with 95% of A, and then B was raised to 28% in 105 min and to 40% in 15 min. Last, the column was washed with 95% of B for 20 min and reequilibrated with 95% of A for 10 min. Peptide masses were analyzed in the Orbitrap cell in full ion scan mode, at a resolution of 120,000, a mass range of mass/charge ratio 350 to 1550, and an AGC target of 4.10^5^. Tandem MS (MS/MS) was performed in the top-speed 3-s mode. Peptides were selected for fragmentation by higher-energy C-trap dissociation with a normalized collisional energy of 27% and a dynamic exclusion of 60 s. Fragment masses were measured in an ion trap in the rapid mode, with an AGC target of 1.10^4^. Monocharged peptides and unassigned charge states were excluded from the MS/MS acquisition. The maximum ion accumulation times were set to 100 ms for MS and 35 ms for MS/MS acquisitions, respectively.

Label-free quantification was done on Progenesis QI for Proteomics (Waters, Milford, MA, USA) in Hi-3 mode for protein abundance calculation. MGF peak files from Progenesis were processed by Proteome Discoverer 2.4 with the Mascot search engine. The Swissprot protein database was typically used for interrogation. A maximum of two missed cleavages was authorized. Precursor and fragment mass tolerances were set to 7 parts per million and 0.5 Da, respectively. The following posttranslational modifications were included as a variable: oxidation (M) and phosphorylation (STY). Spectra were filtered using a 1% false discovery rate using the percolator node.

### UV CRAC

The CRAC protocol used in this study is derived from the work of Granneman *et al.* (*37*) with several modifications as previously described (*36*). Briefly, 2 liters of cells expressing an HTP-tagged version of Rpc160 (the largest subunit of RNAPIII) at the endogenous locus was grown at 30°C to OD_600_ of ~0.6 in CSM-TRP medium (complete amino acid supplement mixture without tryptophan). Cells were cross-linked for 50 s using a W5 UV cross-linking unit (UVO3 Ltd.) and harvested by centrifugation. Cell pellets were washed once with ice-cold 1× phosphate-buffered saline and resuspended in 2.4 ml of TN150 buffer [50 mM tris-HCl (pH 7.8), 150 mM NaCl, 0.1% NP-40, and 5 mM β-mercaptoethanol] per gram of cells in the presence of protease inhibitors (cOmplete EDTA-free Protease Inhibitor Cocktail, Roche). Suspensions were flash-frozen in droplets, and cells were subjected to cryogenic grinding using a Ball Mill MM 400 (five cycles of 3 min at 20 Hz). The resulting frozen lysates were thawed on ice and digested with deoxyribonuclease I (165 U/g of cells) at 25°C for 1 hour to solubilize chromatin and then clarified by centrifugation at 16 krpm for 30 min at 4°C.

RNA-protein complexes were immobilized on M-280 Tosylactivated Dynabeads coupled with rabbit IgGs (10 mg of beads per sample), washed with TN1000 buffer [50 mM tris-HCl (pH 7.8), 1 M NaCl, 0.1% NP-40, and 5 mM β-mercaptoethanol], and eluted by digestion with the TEV protease. RNAs were subjected to partial degradation to reduce their size by adding 0.2 U of RNase cocktail (RNace-IT, Agilent), and the reaction was stopped by the addition of guanidine-HCl to a final concentration of 6 M. RNA-protein complexes were then incubated with Ni–NTA (nitrilotriacetic acid) Sepharose (100 μl of slurry per sample; QIAGEN) overnight at 4°C and extensively washed. Sequencing adapters were ligated to the RNA molecules as described in the original procedure. RNA-protein complexes were eluted with elution buffer containing 50 mM tris-HCl (pH 7.8), 50 mM NaCl, 150 mM imidazole, 0.1% NP-40, and 5 mM β-mercaptoethanol fractionated using a Gel Elution Liquid Fraction Entrapment Electrophoresis (GelFree) system (Expedeon), following the manufacturer’s specifications. The fractions containing Rpc160 were treated with 100 μg of proteinase K, and RNAs were purified and reverse-transcribed using reverse transcriptase Superscript IV (Invitrogen).

The cDNAs were amplified by polymerase chain reaction (PCR) using LA Taq polymerase (Takara), and then the PCR reactions were treated with exonuclease I (200 U/ml; New England Biolabs) for 1 hour at 37°C. Last, the DNA was purified using NucleoSpin columns (Macherey-Nagel) and sequenced on a NextSeq 500 Illumina sequencer.

### Synchronization of cells in G_1_ and analysis by flow cytometry

Two liters of cells were synchronized in the G_1_ phase of the cell cycle by adding 4 mg of α factor. To maintain cells in G_1_, 8 and 4 mg of α factor were subsequently added to the culture after 1 and 2 hours of incubation at 30°C, respectively. Cells were collected and processed 1 hour after the last addition of α factor.

To analyze the DNA content of synchronized cells, 2 ml of culture was collected at different time points, and cells were harvested by centrifugation. Cell pellets were resuspended in 50 mM sodium citrate buffer and treated with RNase A (QIAGEN) for 2 hours at 50°C, followed by proteinase K (Sigma-Aldrich) treatment for 2 hours at 50°C. Cell aggregates were then dissociated by sonication, and 40 μl of cell suspension was incubated with 170 μl of 50 mM sodium citrate buffer containing 0.5 μM SYTOX Green (Invitrogen). Data were acquired on a MACSQuant Analyzer (Miltenyi Biotec) and analyzed with FlowJo software.

### Dataset processing

CRAC reads were demultiplexed using the pyBarcodeFilter script from the pyCRACutility suite (*57*). Next, the 5′ adapter was clipped with Cutadapt, and the resulting insert was quality-trimmed from the 3′ end using Trimmomatic rolling mean clipping (*58*). We used the pyCRAC script pyFastqDuplicateRemover to collapse PCR duplicates using a 6-nt random tag included in the 3′ adapter. The resulting sequences were reverse-complemented with the Fastx reverse complement that is part of the fastx toolkit (http://hannonlab.cshl.edu/fastx_toolkit/) and mapped to the R64 genome with bowtie2 using “-N 1” option. Reads mapping to multiple sites (e.g., most reads mapping to the body of tRNA genes) were attributed randomly to one of the matching sites. Therefore, the RNAPIII signal at those repeated sequences represents the average of all identical regions. Reads shorter than 20 nt were filtered out after mapping, and coverage files were generated and normalized to counts per million (CPM) using the bamCoverage tool from the deepTools package (*59*) using a bin size of 1.

### Bioinformatic analyses

All sequence files and annotations were obtained from Saccharomyces Genome Database (*S. cerevisiae* genome version R64-2-1). T-tracts were annotated by first searching for sequences containing at least four consecutive thymines (for the plus strand) or adenines (for the minus strand) using the unix command line tool *grep* and then generating coordinate files by the *awk* command. The resulting files were then combined into a single BED file (table S7) using BEDOPS suite (*60*) with the *everything* option. For each tRNA gene, the primary terminator was defined as the first T-tract after the 3′ end of the mature tRNA. These primary terminators were identified by comparing the mentioned T-tract annotations and the tRNA annotations with the *closest* tool from BEDTools (*61*). T-tracts falling within the 700-bp region immediately downstream of the primary terminator of each tRNA gene were identified with the BEDTools *intersect* tool and defined as secondary terminators (table S8).

Reads mapped to different classes of RNAs were summarized by BEDTools *coverage*. Metagene analyses of RNAPIII occupancy were performed with deepTools suite (*59*). Strand-specific coverage bigwig files and modified tRNA coordinates (from the 5′ end to the end of the first T-tract) were used as inputs for the *computeMatrix* tool using a bin size of 1 and the scale-regions mode. The matrices generated for each strand were subsequently combined by the *computeMatrixOperations* tool with the *rbind* option and used as inputs for the *plotProfile* tool to create a summary plot. For heatmap analyses, the log_2_ ratio of the RNAPIII signal in the *sen1-3* mutant relative to the WT was calculated by the *bigwigCompare* tool using a bin size of 1 and the corresponding bigwig coverage files as inputs. Matrices were generated and combined as for metagene analyses, and the final matrix was used as the input for the *plotHeatmap* tool. To analyze the correlation between two replicates, the average RNAPIII signal over regions comprising tRNA genes and 500-bp upstream and downstream regions was computed using the *multiBigwigSummary* tool. The resulting tables were used as inputs for the *plotCorrelation* tool to generate scatterplots and calculate the correlation coefficients using the Spearman method.

To annotate tRNA gene RT regions in the WT and the *sen1-3* mutant, we first determined a threshold below which CRAC signal was considered as background signal. To do so, genomic regions corresponding to protein-coding genes, which are transcribed by RNAPII, were divided into 20-bp nonoverlapping windows, and the total signal was computed for each of them using normalized coverage files. The value corresponding to the 95% quantile (value below which 95% of window values fall) was set as threshold. For each tRNA gene, the 1-kb region immediately downstream of the primary terminator was then divided into 20-bp windows with 1-bp overlap, and the RNAPIII CRAC signal was computed for all of them. Contiguous windows with values above the threshold were merged, and, most often, this resulted in a single RT region for each gene. When this was not the case, we manually merged the fragmented regions that were separated by small gaps. Final annotations are provided as BED files in table S9.

The efficiency of transcription termination in the WT and the *sen1-3* mutant was estimated by calculating the RT index defined as the percentage of RNAPIII signal over the RT regions relative to the signal over tRNA gene regions. The total RNAPIII signal at each region was computed with the UCSC *bigWigAverageOverBed* package (http://genome.ucsc.edu) using the aforementioned annotations. Data representation and statistical analyses were performed with R using the *ggplot2* and *plyr* (https://cran.r-project.org/web/packages/plyr/index.html) packages.

### RNA analyses

Yeast cells were grown on 30 ml of YPD medium containing the appropriate additives, depending on the experiment, at 30°C to OD_600_ of 0.3 to 0.6. Cells were harvested by centrifugation, and RNAs were prepared using standard methods. Total RNA (4 μg) was reverse-transcribed by the M-MLV reverse transcriptase (New England Biolabs) following the manufacturer’s specifications and using oligo(dT) and a mixture of random hexamers at 37°C for 45 min. The resulting cDNAs were analyzed by quantitative PCR using the LightCycler 480 SYBR Green I Master reagent (Roche) and LightCycler 480 instrument (Roche) using primers specific for the regions to detect (table S5).

For Northern blot assays, typically 10 μg of total RNA was loaded onto a 10% denaturing polyacrylamide gel and separated by electrophoresis at 20 W for 2 hours. RNAs were then transferred to a nitrocellulose membrane (GE Amersham Hybond-N^+^) using a wet transfer system (Trans-Blot cell, Bio-Rad) at 100 V for 2 hours at 4°C. Membranes were UV–cross-linked and hybridized with the corresponding radioactively labeled probe in commercial buffer (Ultrahyb, Ambion) overnight. For abundant RNA species, we used 5′ end–labeled DNA oligonucleotides as probes, and hybridizations and subsequent washes were performed at 42°C. For RNA species that were very poorly detected using DNA oligonucleotide probes, we used RNA probes generated by in vitro transcription in the presence of α^32^P–uridine 5′-triphosphate using the MAXIscript Kit (Ambion). Hybridization was then performed at 68°C overnight, and the membrane was washed two times for 15 min at 42°C with 2× SSC buffer [30 mM sodium citrate (pH 7.0) and 300 mM NaCl] containing 0.5% SDS and two times for 15 min at 60°C with 0.1× SSC buffer containing 0.1% SDS. After washes, blots were exposed on a PhosphorImager screen and lastly scanned using a Typhoon scanner (GE Healthcare). Images were analyzed using the ImageQuant software (GE Healthcare).

### Protein purification

RNAPIII and RNAPI were purified from *S. cerevisiae* by heparin chromatography, followed by IgG affinity chromatography, and lastly anion exchange using a previously described procedure (*62*) with the following modifications: For cell lysis and equilibration of the heparin column (GE Healthcare), we instead used a buffer containing 250 mM tris-HCl (pH 8), 250 mM ammonium sulfate [(NH_4_)_2_SO_4_], 20% glycerol, 1 mM EDTA, 10 mM MgCl_2_, 10 μM ZnCl_2_, 12 mM β-mercaptoethanol, and a protease inhibitor cocktail [leupeptin (0.3 μg/ml), pepstatin A (1.4 μg/ml), phenylmethylsulfonyl fluoride (170 μg/ml), and benzamidin (330 μg/ml)]. Purified RNAPIII and RNAPI were buffer-exchanged to storage buffer [15 mM Hepes (pH 7.5), 150 mM (NH_4_)_2_SO_4_, and 10 mM DTT], concentrated to 14.9 mg/ml (RNAPIII) and 10.4 mg/ml (RNAPI), snap-frozen in liquid nitrogen, and stored at −80°C.

Full-length Sen1 was overexpressed from pFL vector in insect cells (*Trichoplusia ni*) using an optimized synthetic gene (GeneArt, Life Technologies) and the baculovirus expression system (*63*). Briefly, Hi5 cells (Thermo Fisher Scientific) expressing a C-terminally His_6_-tagged version of Sen1 were harvested by centrifugation and lysed by sonication at 4°C in ice-cold buffer A1 [50 mM Hepes-NaOH (pH 7.75), 600 mM NaCl, 15% (v/v) glycerol, 5 mM β-mercaptoethanol, 10 mM imidazole, and 2 mM MgCl_2_] supplemented with a cocktail of EDTA-free protease inhibitors (Roche). The lysate was clarified by centrifugation at 15,000 rpm for 1 hour at 4°C, filtered through a 0.45-μm filter, and loaded on a 5-ml Protino Ni-NTA agarose prepacked column (Macherey-Nagel), equilibrated with buffer A1. To get rid of nucleic acids bound to Sen1, the column was washed with high-salt buffer B2 [50 mM Hepes-NaOH (pH 7.75), 1 M NaCl, 15% (v/v) glycerol, 5 mM β-mercaptoethanol, and 2 mM MgCl_2_] and equilibrated back to buffer A1. Proteins were eluted using a linear gradient from buffer A1 to B [50 mM Hepes-NaOH (pH 7.75), 200 mM NaCl, 10% (v/v) glycerol, 5 mM β-mercaptoethanol, 400 mM imidazole, and 2 mM MgCl_2_], diluted with two volumes of buffer D1 [50 mM Hepes-NaOH (pH 7.75), 50 mM NaCl, 15% (v/v) glycerol, 2 mM β-mercaptoethanol, and 2 mM MgCl_2_], and then loaded onto two tandem 5-ml heparin HP prepacked columns (GE Healthcare). Sen1 was eluted using a linear gradient from 20 to 100% of buffer B2 containing 50 mM Hepes-NaOH (pH 7.75), 1 M NaCl, 5% (v/v) glycerol, 2 mM MgCl_2_, and 2 mM DTT. Peak fractions were pooled and subjected to size exclusion chromatography using a Superdex 200 16/600 column (GE Healthcare) equilibrated in buffer A3 [50 mM Hepes-NaOH (pH 7.75), 300 mM NaCl, 5% (v/v) glycerol, 2 mM Mg acetate, and, 2 mM DTT]. Last, the fractions of interest were concentrated using an Amicon Ultra-100 centrifugal filter (Millipore), aliquoted, flash-frozen in liquid nitrogen, and stored at −80°C.

### In vitro transcription termination assays

RNAPIII transcription termination assays were performed using the previously described method for RNAPII (*64*) with some modifications. For each reaction, ECs were assembled by annealing 2.5 pmol of 5′-end radioactively labeled RNA primer with 2.5 pmol of template DNA oligo in hybridization buffer [20 mM Hepes-NaOH (pH 7.6), 100 mM NaCl, 12 mM MgCl_2_, and 10 mM DTT]. Subsequently, the RNA:DNA hybrids were incubated with 2 pmol of highly purified RNAPIII in transcription reaction buffer (TRB) [20 mM Hepes-NaOH (pH 7.6), 60 mM (NH_4_)_2_SO_4_, 10 mM MgCl_2_, 10% glycerol, and 10 mM DTT] at 20°C for 10 min at 550 rpm. Next, 5 pmol of 5′-end biotinylated nontemplate DNA was added to the mixture and incubated at 20°C for 10 min with shaking. The resulting ternary ECs were mixed with streptavidin beads (Dynabeads MyOne Streptavidin T1 from Invitrogen; 10 μl of slurry per reaction) prewashed four times with TRB buffer containing 0.1% Triton X-100 and then incubated at 20°C for 30 min with gentle shaking. After binding, the beads were washed with one volume of TRB containing 0.1% Triton X-100, then with one volume of TRB containing 250 mM (NH_4_)_2_SO_4_, and lastly with one volume of TRB. After washes, beads were resuspended in 13 μl of TRB buffer. The reaction was started by adding 7 μl of nucleotide mixture (1 mM each in TRB buffer) and incubating at 28°C for 10 min and then stopped by the addition of 1 μl of 0.5 M EDTA. Beads and supernatant fractions were then collected separately. RNAs in the supernatant were ethanol-precipitated and resuspended in 10 μl of loading buffer containing 1× tris-borate–EDTA (TBE) and 8 M urea and incubated at 95°C for 3 min before loading onto a 10% denaturing polyacrylamide gel. To isolate RNAs from beads, 10 μl of loading buffer was added to the beads and boiled at 95°C for 3 min and then recovered supernatants as “bead fractions.” Last, samples were subjected to 10% denaturing polyacrylamide gel electrophoresis (PAGE), running for 1 hour at 40 W in 1× TBE buffer. Gels were exposed on a PhosphorImager screen overnight at −80°C, and screens were scanned using a Typhoon scanner (GE Healthcare). Images were analyzed using the ImageQuant software (GE Healthcare).

## Notes

View/request a protocol for this paper from *Bio-protocol*.
