# Supplementary material for: An integrated model for termination of RNA polymerase III transcription
Source: Sci Adv. 2022 Jul 13;8(28):eabm9875. doi: 10.1126/sciadv.abm9875 (PMC9278858; doi:10.1126/sciadv.abm9875)
Supplement: Supplementary file 1 — Figs. S1 to S8 Table S6 [file sciadv.abm9875_sm.pdf]

Science Advances
AAAS

Supplementary Materials for

An integrated model for termination of RNA polymerase III transcription

Juanjuan Xie *et al.*

Corresponding author: Domenico Libri, domenico.libri@igmm.cnrs.fr; Odil Porrua, odil.porrua@igmm.cnrs.fr

Sci. Adv. **8**, eabm9875 (2022)
DOI: 10.1126/sciadv.abm9875

The PDF file includes:

Figs. S1 to S8
Table S6

Other Supplementary Material for this manuscript includes the following:

Tables S1 to S5, S7 to S9

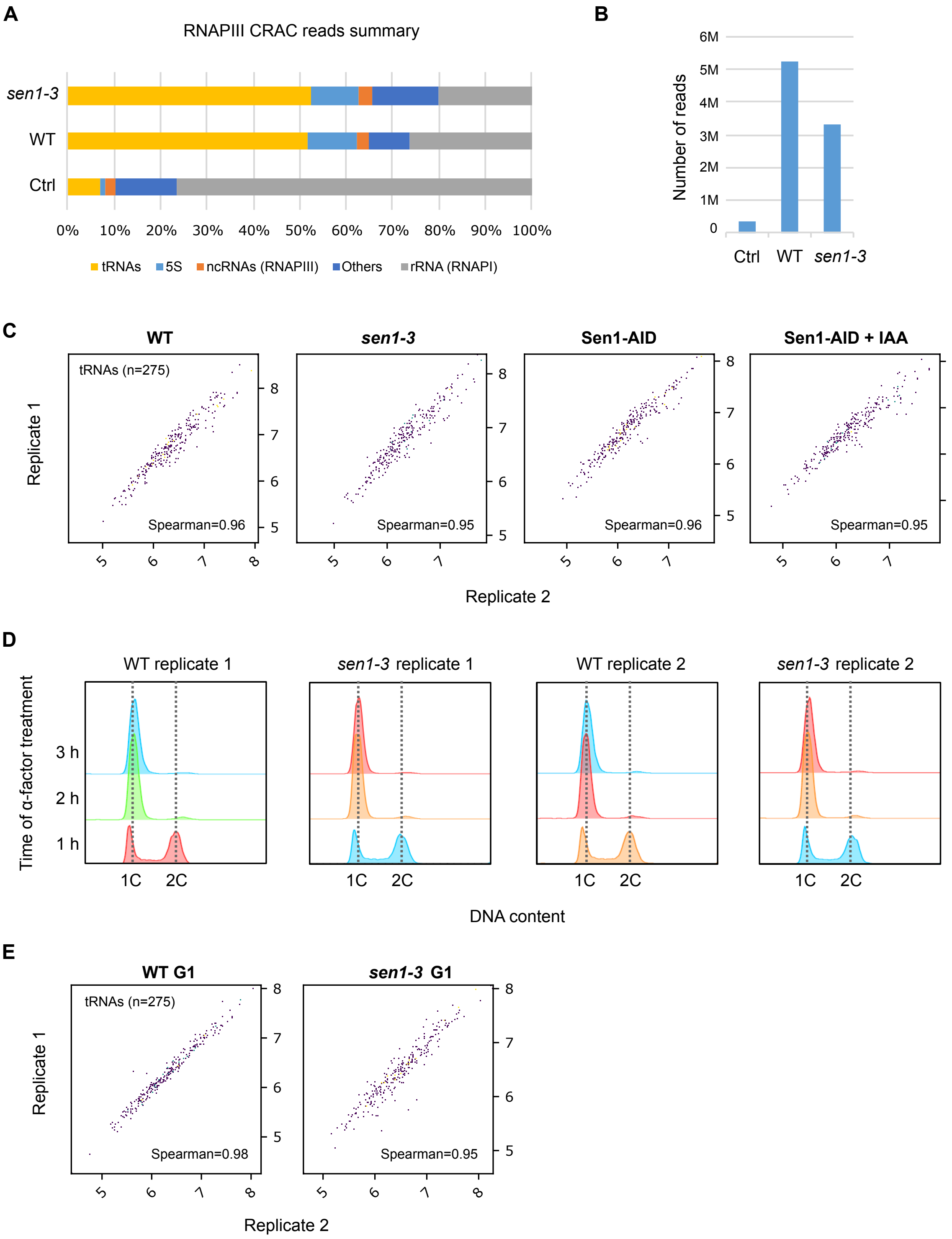

Fig. S1: Complementary analyses validating CRAC experiments in Fig.s 2 and 3.

(A) Comparison of the reads distribution among different genomic regions in crosslinked samples (WT and *sen1-3*) relative to the un-crosslinked control (Ctrl) in a typical RNAPIII CRAC experiment. The "others" category corresponds to RNAPII genes and intergenic regions. Note that tRNA read-through regions are included in this category and the larger proportion of reads in this

group in the *sen1-3* mutant could be due to the observed increased RNAPIII presence at those regions. **(B)** Plot representing the number of mapped reads obtained in a typical CRAC experiment in the different samples. Note that the number of reads in cross-linked samples is at least one order of magnitude higher than in the un-crosslinked control (Ctrl). "M" denotes millions. **(C)** Scatter plots showing the high correlation between the two biological replicates of each condition/strain for the CRAC experiments showed in Fig. 2. **(D)** Analysis of DNA copy-number for samples in Fig. 3D-F by flow cytometry. 1C and 2C corresponds to 1 and 2 copies of the genome, respectively. Cultures were used for CRAC analyses after 3h of treatment with α-factor (see methods). **(E)** Correlation plots for the two biological replicates of samples used in experiments in Fig. 3D-F.

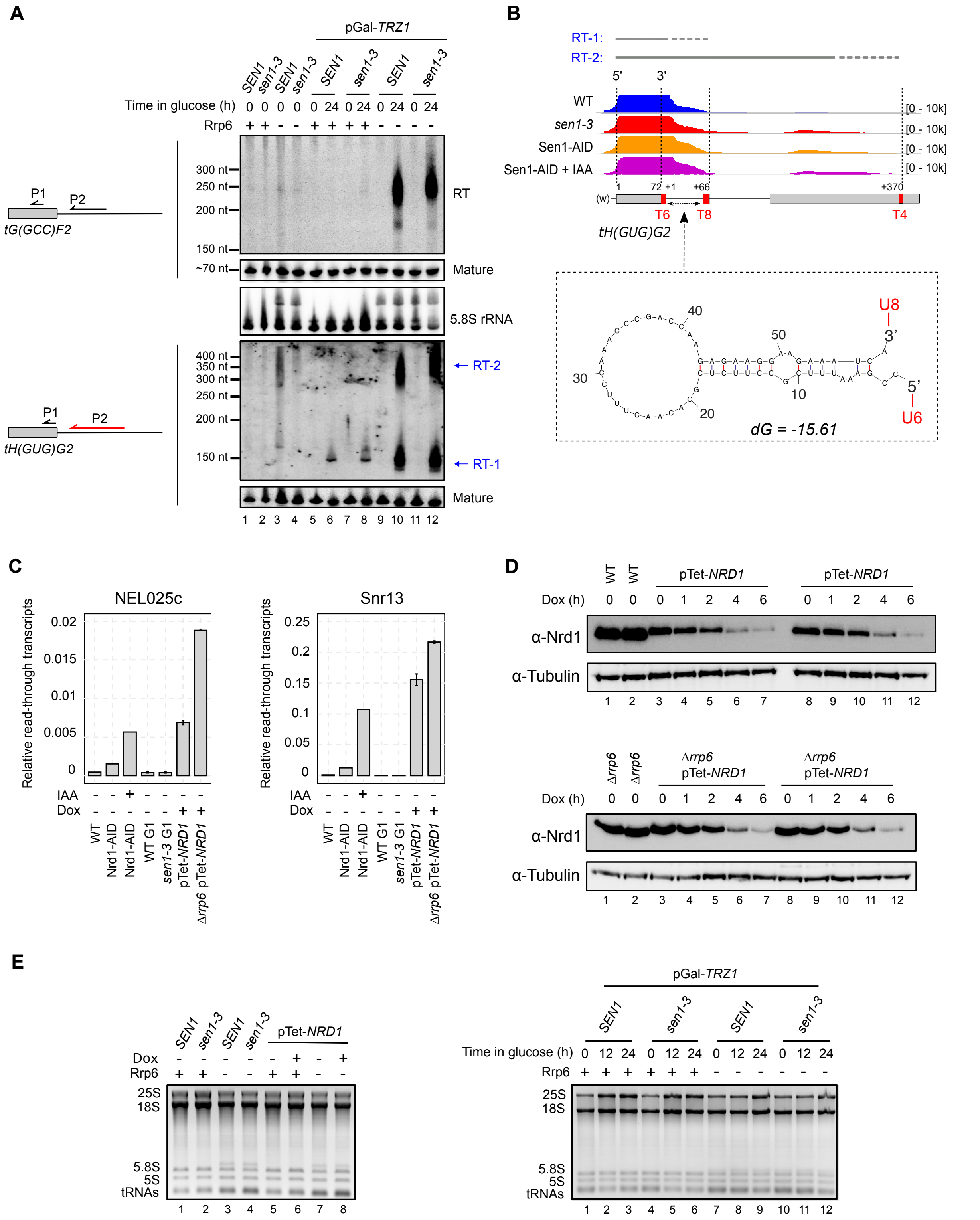

Fig. S2: Experiments related to Fig.s 2 and 3.

(A) Northern blot analysis of transcripts derived from two different tRNA genes in the indicated backgrounds. In pGal-*TRZ1* strains, the essential gene *TRZ1* is expressed from the *GAL1* promoter

and the different strains are either grown on galactose for the whole experiment (t=0) or shifted to medium containing glucose for 24h to repress *TRZ1*. Schemes on the left indicate the approximate position of the probes (P1 and P2) used for the detection of the different RNA species (RT, for read-through, and mature tRNA). The RNA probe is indicated in red, while DNA oligonucleotide probes are indicated in black (more details in table S5). The 5.8S rRNA is used as a loading control. **(B)** IGV screenshot of the region around the *tH(GUG)G2* gene indicating the position of different T-tracts found and the two major groups of RT transcripts detected by northern blot (**A**, bottom blot). Note that the fact that we detect multiple bands for each termination region could be due to the existence of several termination sites and/or the presence of heterogeneous poly(A) tails. The structure of the RNA between the T6 and the T8 T-tract predicted by the mFold software of the UNAFold package (http://www.unafold.org/) is shown on the bottom. **(C)** Analysis of transcription termination defects at two well-characterized NNS-targets (the CUT NEL025c and the snoRNA gene *SNR13*) in the indicated strains. RNAs where prepared from the same cultures used for CRAC experiments and northern blot analyses in Fig.s 2 and 3. Typical read-through transcripts resulting from inefficient termination by the NNS-complex where detected by RT-qPCR with oligonucleotides listed in table S5. Values in the “y” axis correspond to the abundance of RT RNAs relative to the levels of the *ACT1* mRNA. **(D)** Western blot analysis of Nrd1 depletion by incubation of pTet-*NRD1* strains (2 biological replicates) with doxycycline (Dox) for the indicated times. Tubulin detection was used as a loading control. **(E)** Native agarose gels showing total RNA levels in the indicated samples stained with ethidium bromide.

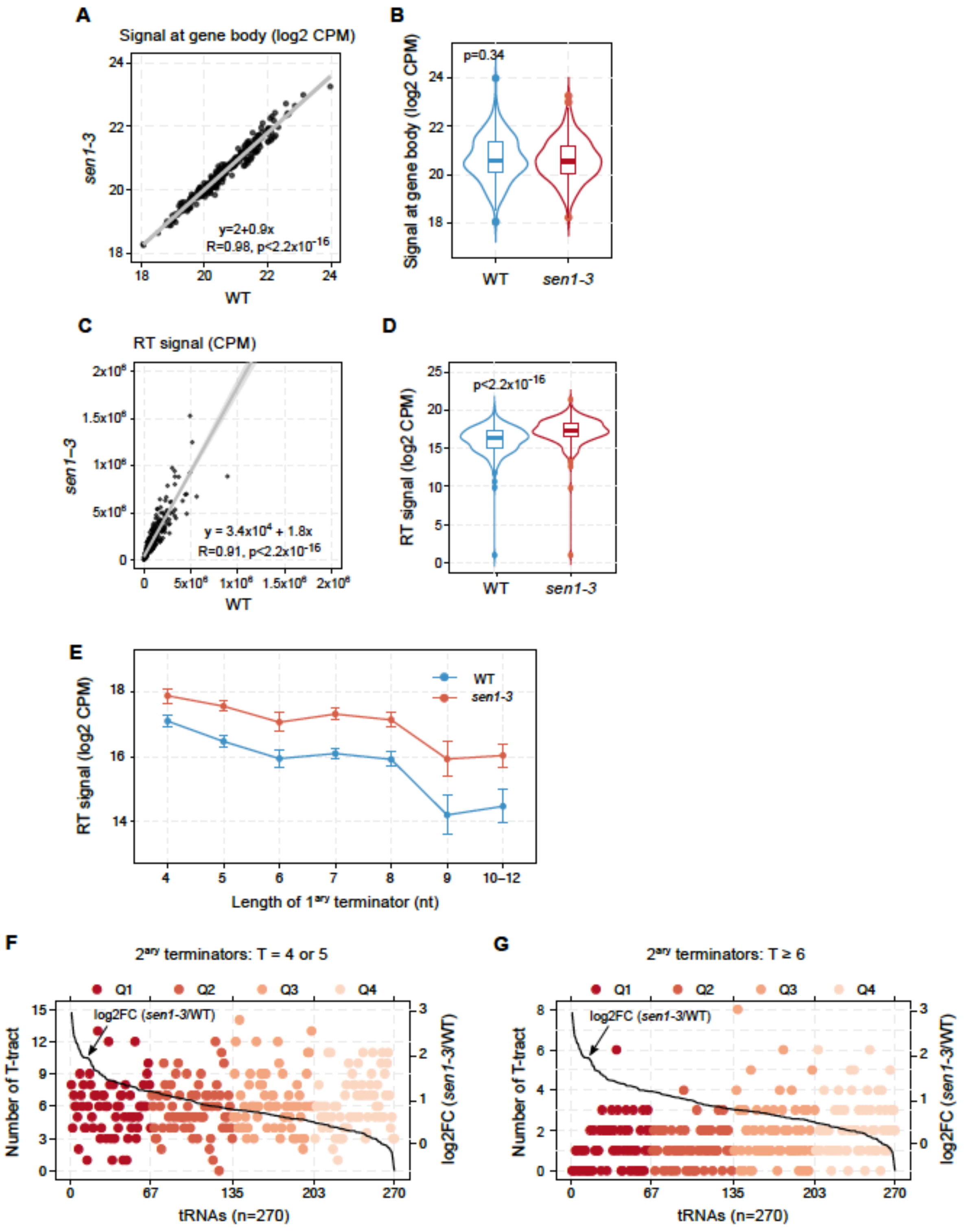

Fig. S3: Complementary analyses related to Fig. 4.

(A) and (**B)** Comparison of the RNAPIII CRAC signal at the body of tRNA genes (i.e. the region from the 5' end of the mature tRNA to the first T-tract) in the WT and in the *sen1-3* mutant. (**A)** Dispersion plot where "R" corresponds to Pearson's correlation coefficient and *p* is the associated

p-value. **(B)** Violin plot where p corresponds to the p-value calculated by the Wilcoxon test. **(C)** and **(D)** Comparison of the RNAPIII CRAC signal at the RT region of tRNA genes in the WT and in the *sen1-3* mutant. **(C)** Dispersion plot where "R" corresponds to Pearson's correlation coefficient and p is the associated p-value. Three outliers are not shown. **D)** Violin plot where p corresponds to the p-value calculated by the Wilcoxon test. **(E)** Analysis of the RT signal at tRNA genes grouped according to the length of their primary terminator in either the WT or the *sen1-3* mutant. Data points correspond to the average values whereas error bars denote the standard error. **(F)** and **(G)** Representation of the number of T-tracts of indicated lengths located in the 700 bp region downstream of the primary terminator of each tRNA gene. Data points are coloured according to the quartile (Q) they belong to. Quartiles are defined according to the log2 FC of the RNAPIII signal in *sen1-3* relative to the WT at the same region, which provides an estimation of the dependency on Sen1 for termination. Thus, Q1 includes the most Sen1-dependent tRNA genes.

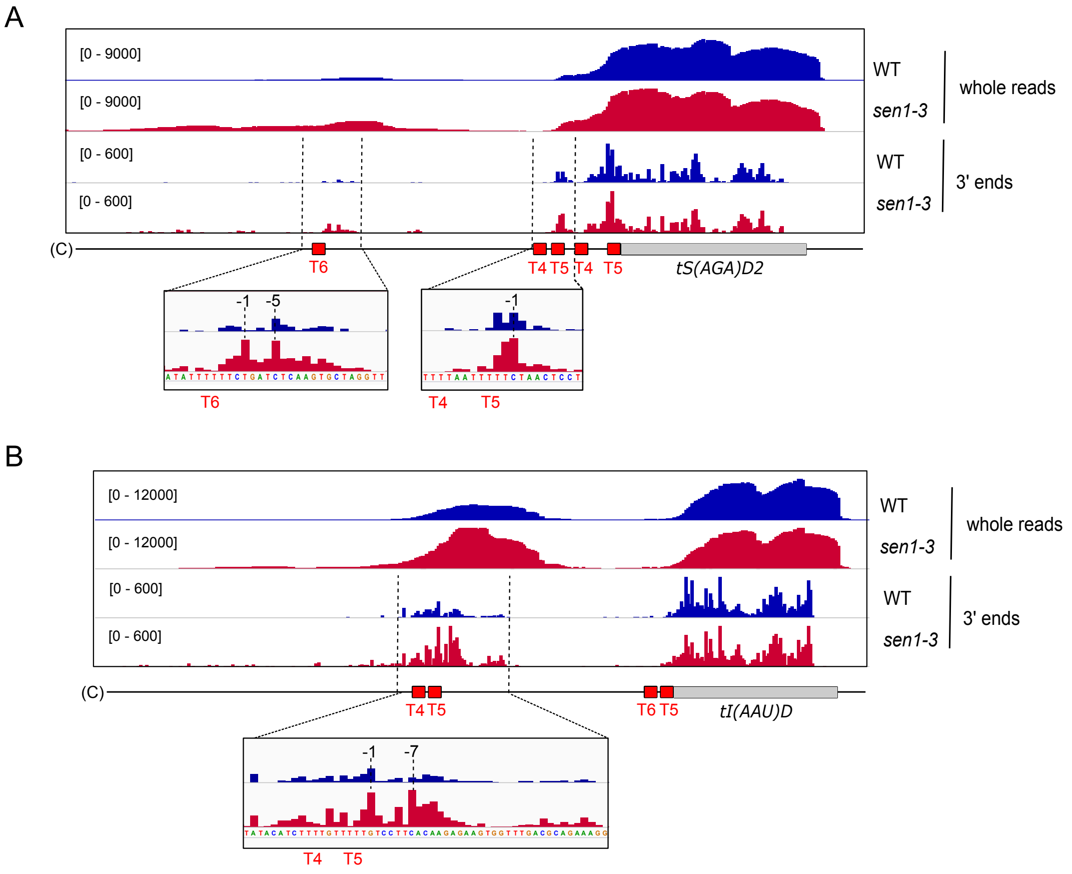

Fig. S4: Examples of tRNA genes illustrating the role of Sen1 in enhancing secondary termination. IGV screenshots showing the distribution of RNAPIII CRAC signal in the WT and the *sen1-3* mutant with zoom-in views of the main regions where RNAPIII accumulates in the mutant. The 3' ends datasets provide the position of individual RNAPIIIs with single-nucleotide resolution. Most accumulation is observed just upstream of or at the first Ts of secondary weak terminators, suggesting impaired RNAPIII release by Sen1 at these sites. The indicated coordinates correspond to the position relative to the beginning of the nearest downstream T-tract.

1) No hairpin and T4, T5, T6, T9 or T12 terminators (figures 5, and 6)

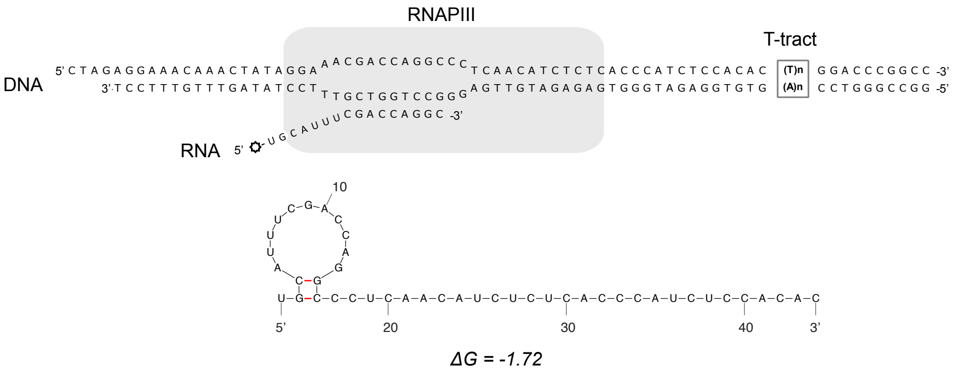

2) Hairpin (HP) and T4, T6 or T12 terminators (figure 7E-F)

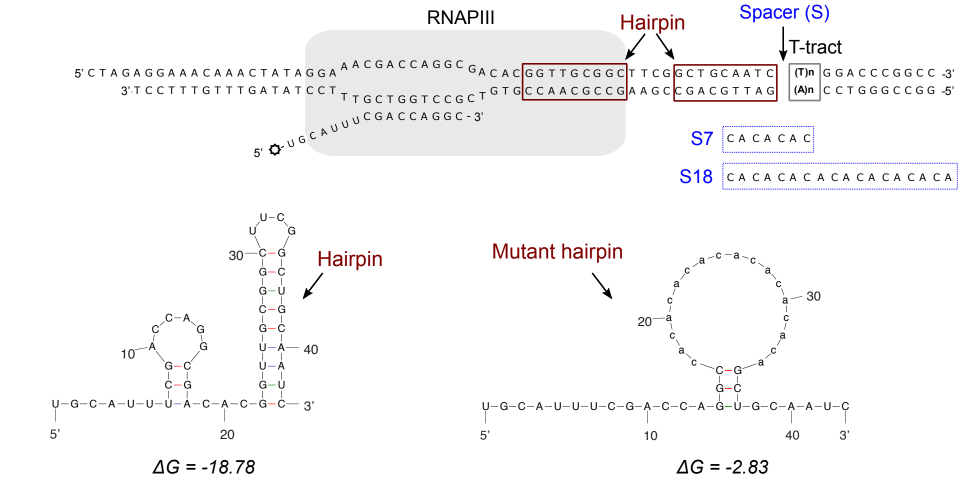

3) Hairpin (HP) - A-less cassette - T4 terminator - A-tract (figure 7E)

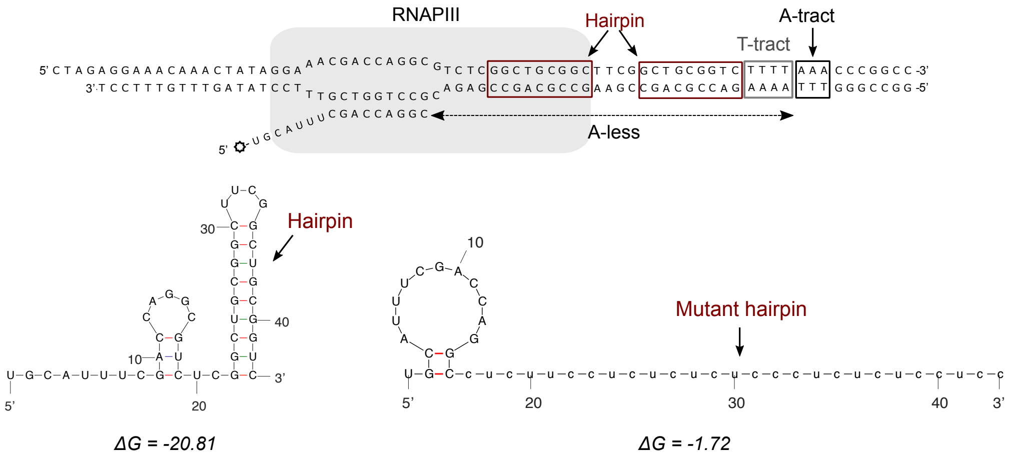

Fig. S5: Sequence of transcription templates and predicted structure of the different transcribed RNAs in *in vitro* transcription termination assays.

The sequence of the wild-type version of each template are indicated in the schemes. The mutant version of the transcribed RNAs is shown together with the wild-type version under the corresponding scheme. The sequence of the spacers (S) correspond to the non-template strand. RNA structure predictions and ΔG calculation for each structure were performed with the mFold software.

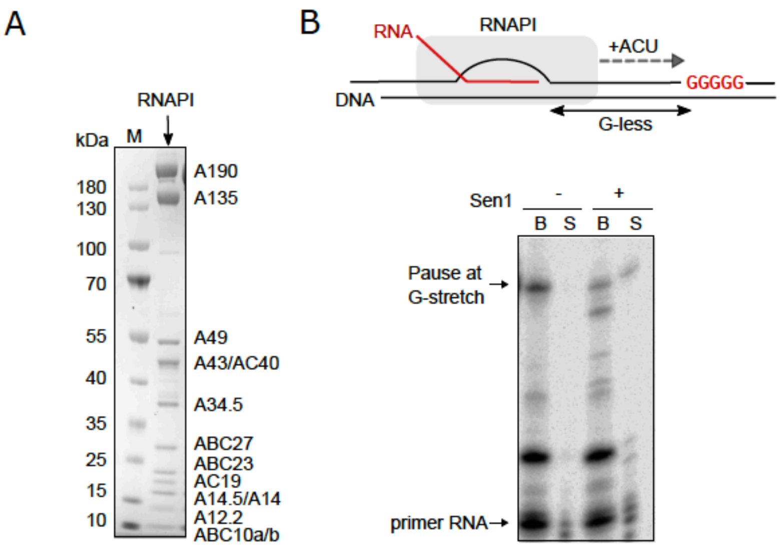

Fig. S6: Sen1 can promote the release of paused RNAPIs *in vitro*.
(A) SDS-PAGE analysis of the RNAPI preparation used in these assays. **(B)** IVTT assay performed on templates containing a G-less cassette followed by a run of Gs to promote stalling of RNAPI at the first G in the absence of guanine in the reaction. Top: scheme of the transcription templates. Bottom: Denaturing PAGE analysis of transcripts from one out of two independent IVTT assays, which produced very similar results.

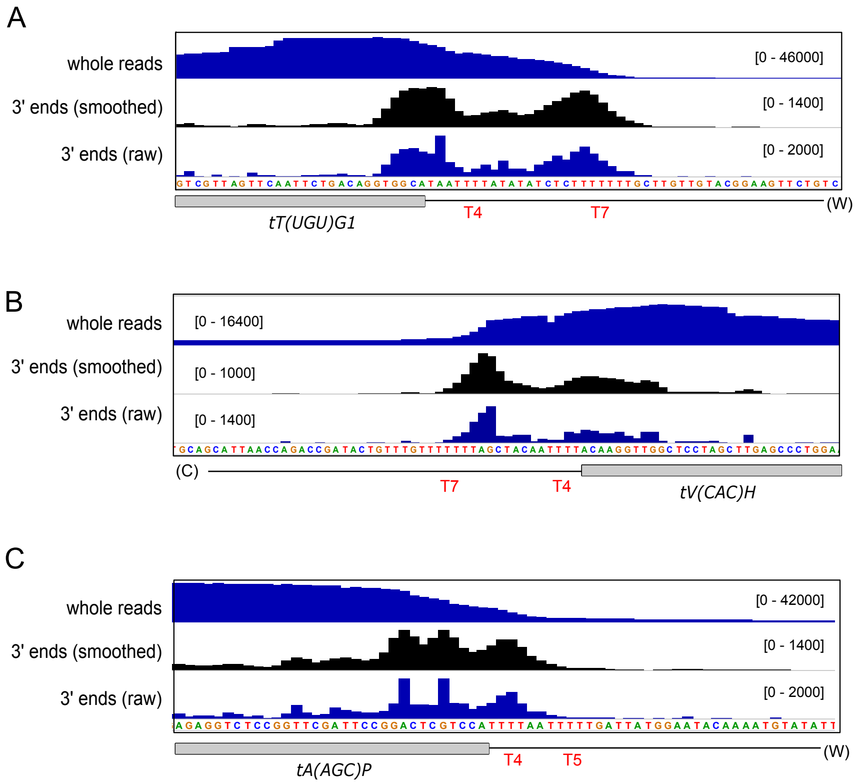

Fig. S7: Examples of tRNA genes harbouring a T4 primary terminator.
(A) and **(B)** Cases where the T4 terminator seems to function autonomously to promote moderate levels of termination. The 3' end datasets provide accurately the position of individual RNAPIIIs, and therefore the location of pausing sites. The decrease in the whole reads RNAPIII signal within the T4 sequence despite the presence of very strong pausing at close downstream T-tracts supports the idea that a fraction of RNAPIIIs terminate at the T4 terminator. **(C)** Example where the T4 sequence seems to function in combination with the downstream T5 T-tract, as suggested by the strong termination observed at these sequences compared to **(A)** and **(B)**, where the T4 sequence is further away from other T-tracts. The pausing pattern, with accumulation of RNAPIIIs at the first 3 thymidines of the T4 sequence rather resembles the pattern observed for long T-tracts (e.g. T9 terminators) *in vitro*. Datasets correspond to the WT strain.

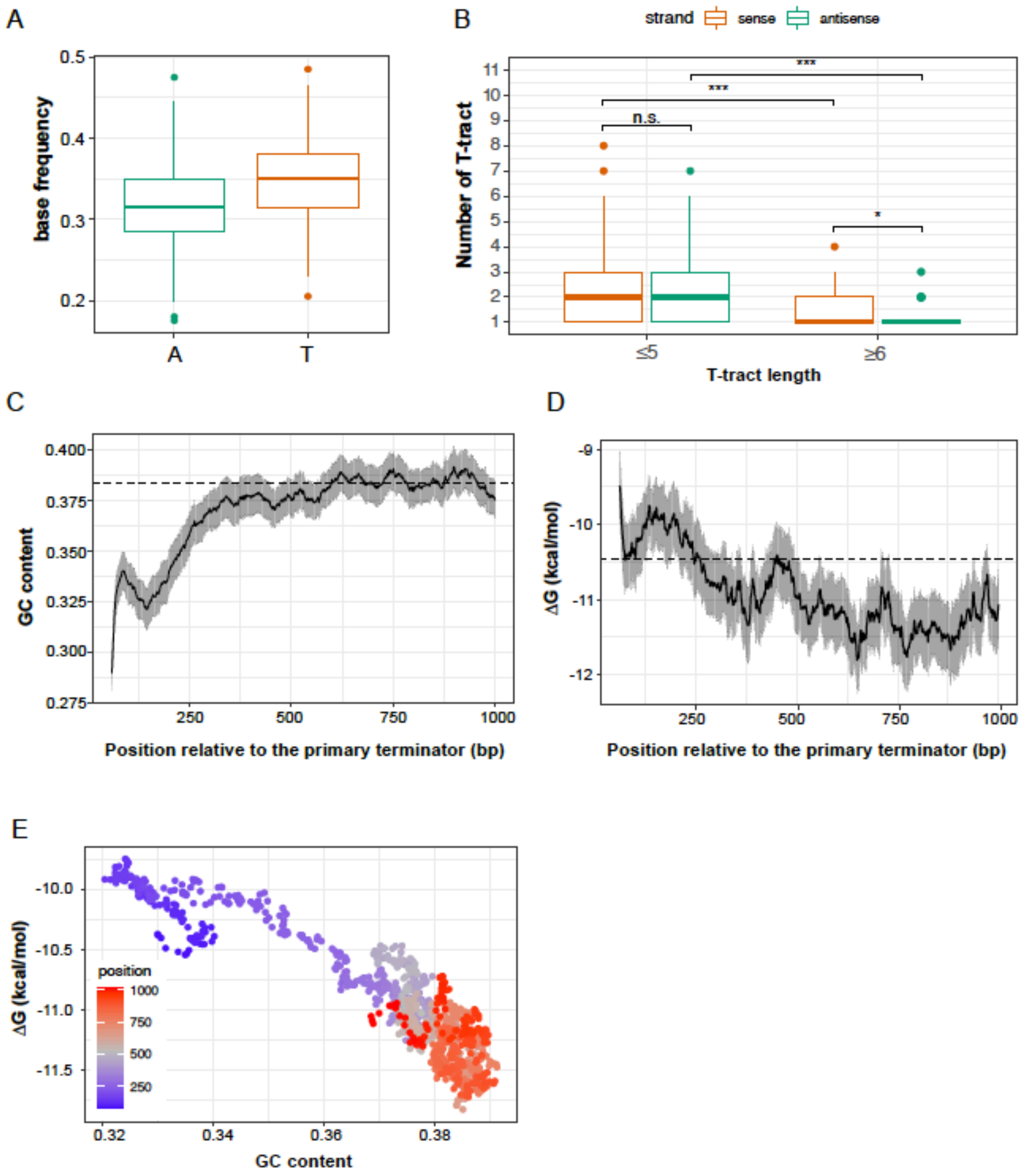

Fig. S8: Analysis of T-tracts and RNA structures at regions of secondary termination.
(A) Analysis of the frequency of A and T nucleotides in the 200 bp region downstream of the primary terminator of tRNA genes. **(B)** Comparison of the number of weak ($T \leq 5$) or strong ($T \geq 6$) terminators at the 200 bp region downstream of the primary terminator of tRNA genes in the sense orientation versus the antisense orientation relative to transcription. Statistical significance was calculated using a Wilcoxon rank sum test. n.s indicates no significant difference between the compared groups whereas * denotes a p-value ≤ 0.05 and *** a p-value ≤ 0.001. **(C)** Analysis of

the GC content (fraction of G and C nucleotides) of regions downstream of the primary terminator of tRNA genes. Values were calculated for 60 bp sliding windows. The black line corresponds to the average value whereas the grey zone represents the 95% confidence interval of the average value. A dashed line indicates the average value for the whole genome. **(D)** Analysis of the Gibbs free energy (ΔG) as a proxy for the propensity of the transcribed regions to form secondary structures. We plotted values calculated for 65 bp sliding windows published in Turowski et al. (*38*). A dashed line indicates the average ΔG value for the whole genome. **(E)** Combined representation of the GC content and the ΔG of the different regions coloured according to the distance from the primary terminator. Closer regions (blue) tend to be less GC-rich and less structured while further regions (red) tend to be more GC-rich and structured.

Number	Name	Genotype	Source/ reference
DLY671	BMA	as W303, *Δtrp1*	(*15*)
DLY1152	*Δrrp6*	as W303, *rrp6::URA3*	This work
DLY1605	*pTet-NRD1*	as BMA, *pTet::FLAG::NRD1*	This work
DLY1626	*pTet-NRD1, Δrrp6*	as BMA*, pTet::FLAG::NRD1, rrp6::KAN*	This work
DLY1656	P_{GAL1} *TAP-SEN1*	as BMA, *TRP1::Pgal::TAP::SEN1*	(*15*)
DLY2692	P_{GAL1} *TAP-sen1ΔNter*	as BMA, *TRP1::Pgal::TAP::sen1ΔNter (Δ1-975)*	(*19*)
DLY3171	Sen1-TAP	as W303, *SEN1::TAP::KAN*	(*33*)
DLY3173	*sen1-3*-TAP	as W303, *sen1W773A,E774A,W777A::TAP::KAN*	(*33*)
DLY3197	*sen1-3*	as W303, *sen1W773A,E774A,W777A*	This work
DLY3246	*sen1-3, Δrrp6*	*as W303, sen1W773A,E774A,W777A rrp6::URA3*	This work
DLY3262	Rpc160-HTP	as BMA, *RPC160::HTP::TRP1*	This work
DLY3265	Rpc160-HTP, *sen1-3*	as W303, RPC160::HTP::TRP1, *sen1W773A,E774A,W777A*	This work
DLY3343	Rpc160-HTP, Sen1-AID	as BMA, *RPC160::HTP::TRP1, SEN1-AID::KAN::OsTIR1*	This work
DLY3377	Rpc160-HTP, Nrd1-AID	as BMA, *RPC160::HTP::TRP1*, *NRD1-3Flag-AID::KAN::OsTIR1*	This work
DLY3462	GAL::HA-Trz1	*GAL-HA-Trz1::KAN*	This work
DLY3463	GAL::HA-Trz1, sen1-3	GAL-HA-Trz1::KAN, *sen1W773A,E774A,W777A*	This work
DLY3464	GAL::HA-Trz1, *Δrrp6*	*GAL-HA-Trz1::KAN, rrp6::URA3*	This work
DLY3465	GAL::HA-Trz1, *Δrrp6,* sen1-3	*GAL-HA-Trz1::KAN, rrp6::URA3, sen1W773A,E774A,W777A*	This work

Table S6: Yeast strains used in this study.
